# Ru(II)‐Catalyzed Transfer Vinylation of Alcohols

**DOI:** 10.1002/cssc.202501279

**Published:** 2025-10-17

**Authors:** Saša Opačak, Sergey Tin

**Affiliations:** ^1^ Catalysis for sustainable processes Leibniz‐Institut für Katalyse Albert‐Einstein‐Straße 29a 18059 Rostock Germany

**Keywords:** alcohols, recycling, ruthenium, transfer vinylation, vinyl ethers

## Abstract

Ruthenium catalysts are investigated for the transfer vinylation of alcohols using alkyl vinyl ethers as the vinylating agents. Bis(2‐methylallyl)(1,5‐cycloocta­diene)ruthenium(II) is found to perform the reaction efficiently on many primary alcohols and affords average yields in the transfer vinylation of secondary alcohols. A recycling experiment carried out shows that all reaction components can be separated and that the isolated ethyl vinyl ether can be reused as the vinylating agent without detrimental effect.

## Graphical abstract

The first example of Ru‐catalyzed transfer vinylation of alcohols is reported. The reaction uses ethyl vinyl ether as the vinylating agent and solvent, only requiring the catalyst and substrate as additional components. This protocol has been successfully applied to the vinylation of primary and secondary alcohols. The procedure has been scaled to 12.6 g enabling the recycling of organic compounds.

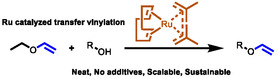

## 1 Introduction

Vinyl ethers are industrially relevant molecules that can be used for the preparation of fine chemicals and polymers.^[^ ^1^, ^2^, ^3^ ^]^ Alcohol groups, which are widely abundant in renewable molecules,^[^ ^4^, ^5^, ^6^ ^]^ can be used for the preparation of vinyl ethers. Reported procedures for the synthesis of simple alkyl vinyl ethers or vinyl acetate from renewable resources allow the production of 100% biobased vinyl ethers.^[^ ^7^, ^8^, ^9^ ^]^ In addition, catalytic transfer vinylation using alkyl vinyl ethers has other advantages: it avoids the generation of stoichiometric amounts of salts, the use of expensive reagents or excess of alcoholic substrates. Up to date, there are only four transition metals reported as catalysts for this transformation.^[^ ^10^ ^]^ Ir catalysts exclusively perform this reaction using vinyl carboxylates as the vinylating agents.^[^ ^11^, ^12^, ^13^, ^14^ ^]^ Hg, Pd, and Au catalysts were shown to perform the reaction using vinyl ethers (usually alkyl vinyl ethers), affording high yields of the products.^[^ ^10^ ^]^ The usage of mercury compounds has severe drawbacks from a safety point of view due to the very high toxicity of this metal. Gold is an expensive noble metal, which also requires 2 mol% catalyst loading and a silver‐containing additive for the efficient transfer vinylation of alcohol groups.^[^ ^15^ ^]^ While palladium catalysts show excellent performance in the reaction,^[^ ^16^, ^17^ ^]^ Pd is an expensive noble metal, which also often requires the presence of air (or Cu(OAc)_2_) to perform the reaction.^[^ ^18^ ^]^ Although running reactions under aerobic conditions can often be an advantage, it can be a limitation if the substrate contains air‐sensitive groups. Another disadvantage appears during scale‐up, as large volumes of volatile ethers and oxygen can form a potentially explosive vapor. In this study, we show the first known example of ruthenium‐catalyzed transfer vinylation of alcohols using alkyl vinyl ethers as vinylating agents. Ru is cheaper than Pd or Au, and the protocol does not require the use of an additional solvent or other additives, employing only the catalyst, alcohol, and the vinylating agent. In a larger‐scale experiment, it was shown that all organic components (ethyl vinyl ether, ethanol, and the newly formed vinyl ether) can be separated and that the ethyl vinyl ether can be reused for alcohol vinylation without any negative impact on product yield.

## 2 Results and Discussion

### 2.1 Testing Different Catalysts and Optimizing the Reaction Conditions

**Figure 1:**
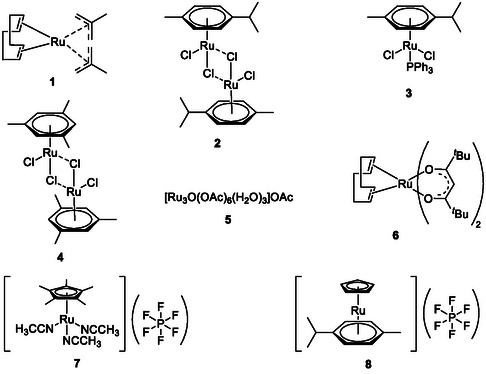
Ru complexes investigated in the transfer vinylation of benzyl alcohol using butyl vinyl ether.

Initially, various catalysts were tested for the transfer vinylation of alcohols with vinyl acetate or alkyl vinyl ethers as the vinyl group sources. After extensive screening of different metal catalysts for the transfer vinylation in the presence of vinyl acetate, in search of an alternative to known Ir catalysts,^[^ ^11^, ^12^, ^13^, ^14^ ^]^ no relevant conversion was ever observed (see Supporting Information 7). Therefore, *n*‐butyl vinyl ether was chosen as the vinylating agent instead. No additional solvent was used as the vinylating agent itself is sufficient to dissolve all the reaction components. While most transition metals afforded either no or only small amounts of the desired product (see Supporting Information 3.1 for details), several Ru complexes (shown in **Figure** **1**) have shown conversions to the desired products above 60% (**Table** **1**, entries 1, 2, and 4).

**Table 1:** Catalytic transfer vinylation of benzyl alcohol using *n*‐butyl vinyl ether as the vinylating agent.

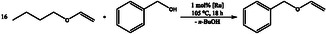			
Entrya)	Ru catalyst	Additional reagents	Benzyl vinyl ether [%]b)
1	**1**	–	66
2	**1**	Et_3_N, 10 mol%	63
3	**2**	–	37
4	**2**	Et_3_N, 10 mol%	62
5	**3**	–	43
6	**3**	Et_3_N, 10 mol%	50
7	**4**	–	33
8	**4**	Et_3_N, 10 mol%	55
9	**5**	–	41
10	**5**	Et_3_N, 10 mol%	49
11	**6**	–	>1
12	**6**	Et_3_N, 10 mol%	>1
13	**7**	–	>1
14	**7**	Et_3_N, 10 mol%	1
15	**8**	–	>1
16	**8**	Et_3_N, 10 mol%	>1
17	[Ru(COD)Cl_2_]_*n*_	–	29
18	[Ru(COD)Cl_2_]_*n*_	Et_3_N, 10 mol%	10
19	[Ru(C_6_H_6_)Cl_2_]_2_	–	26
20	[Ru(C_6_H_6_)Cl_2_]_2_	Et_3_N, 10 mol%	34
21c)	**1**	–	37
22d)	**1**	–	60
23e)	**1**	–	67
24f)	**1**	–	72
25g)	**1**	–	69

a) General conditions: benzyl alcohol (1 mmol), butyl vinyl ether (16 mmol), ruthenium catalyst (1 mol%), hexadecane (50 μL) as the internal standard, 105 °C, 18 h.

b) Conversion was determined by GC (gas chromatography) using hexadecane as the internal standard.

c) Reaction loaded under air.

d) Eight equivalents of *n*‐butyl vinyl ether was used.

e) 2 mol% of Ru used.

f) Reaction time 64 h.

g) 120 °C.

Complex **1** was investigated further as it afforded the best yield (Table 1, entries 1–20) and did not require any additional base. While Pd catalysts are known to operate better under air than under an inert atmosphere in the transfer vinylation of alcohols,^[^ ^18^ ^]^ it is clearly not the case for Ru complex **1** (entry 21). Reducing the volume of the vinylating agent two times results in the reduction of the yield (entry 22). Increasing catalyst loading, prolonging reaction time, or increasing the reaction temperature did not result in any major effect on the product yield (entries 23–25), which might indicate that the equilibrium between *n*‐butyl vinyl ether and the desired product is reached.

**Scheme 1:**
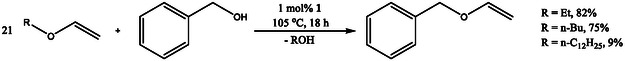
Performance of different alkyl vinyl ethers in transfer vinylation of benzyl alcohol.

**Scheme** **1** shows the vinylating agent substituent effect. Clearly, vinyl ethers with shorter alkyl chains afford higher yields of the desired product. Thus, further optimization was performed using ethyl vinyl ether as the vinylating agent.

Investigation of the optimal ratio of vinylating agent to substrate in catalytic transfer vinylation of benzyl alcohol was performed (**Table** **2**). Using 21 equivalents of ethyl vinyl ether affords benzyl vinyl ether in 82% yield. Using more vinylating agent does not improve the result, as the yields obtained in entries 1 and 2 are within experimental error. However, reducing the amount of vinylating agent has a clear impact on the product yield (entries 2–5). Attempts to reduce the catalyst loading resulted in the reduction of product yield (entry 6). Prolongation of the reaction time with low catalyst loading does not afford a higher yield of the desired product, which indicates that the catalyst deactivates (entries 6 and 7). The addition of triethyl amine does not improve the yield of the product (entry 8). Palladium catalysts bearing acidic counter‐ions benefit from the presence of base,^[^ ^16^ ^]^ as well as some Ru complexes from Table 1. An acidic environment could play a disadvantageous role in this reaction, as vinyl ethers can form acetals with the alcohol present,^[^ ^17^ ^]^ or polymerize.^[^ ^19^ ^]^ However, since catalyst **1** does not bear any acidic counter‐ions, the addition of base to the transfer vinylation reaction is not necessary.

**Table 2:** Optimization of reaction conditions using catalyst **1** and ethyl vinyl ether as the vinylating agent.

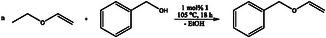		
Entry^a^^)^	Vinylating agent, n	Benzyl vinyl ether [%]^b^^)^
1	42	83
2	21	82
3	17	78
4	15	77
5	13	71
6^c^^)^	21	50
7c), d)	21	50
8c), d), e)	21	42

a) General conditions: benzyl alcohol (1 mmol), ethyl vinyl ether, ruthenium catalyst **1** (1 mol%), hexadecane (50 μL) as the internal standard, 105 °C, 18 h.

b) Conversion was determined by GC using hexadecane as the internal standard.

c) 0.2 mol% of **1** used.

d) Reaction time 64 h.

e) 10 mol% of Et_3_N was added.

### 2.2 Substrate Scope

**Scheme 2:**
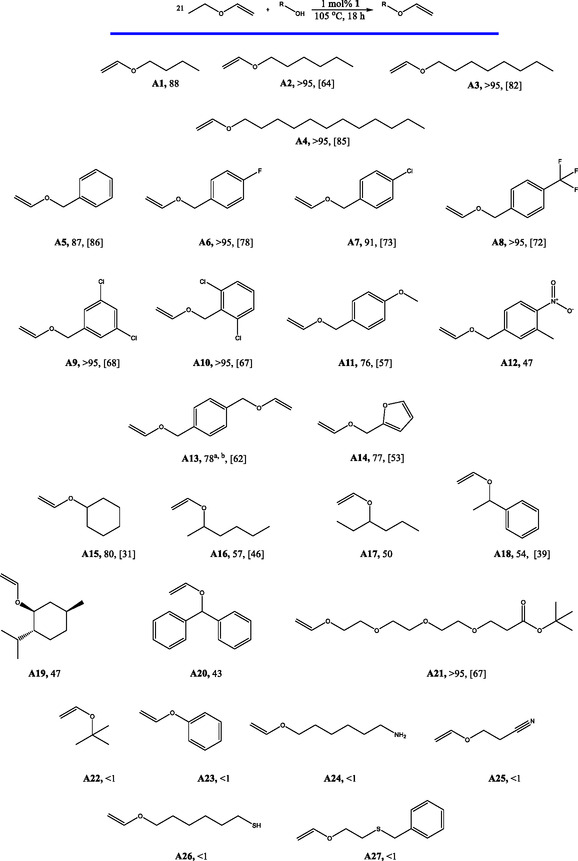
Transfer vinylation of different alcohols by ethyl vinyl ether in the presence of catalyst **1**. GC conversion to the product measured with an internal standard, [isolated yield]. a) 1 mol% of **1** and 21 eq. of ethyl vinyl ether per OH group used, b) total alcohol group conversion to vinyl group.

Having the optimized conditions shown in Table 2, entry 2, the scope and limitations of the method were tested on different substrates (**Scheme** **2**). All simple linear aliphatic primary alcohols apart from butanol (which converts to product **A1**) afforded >95% conversions to the desired vinyl ethers. This is also consistent with the experiment shown in Scheme 1, i.e., it is clear that in the presence of catalyst **1**, the preferred equilibrium lies on the side of the heavier vinyl ethers for linear aliphatic alcohols. Isolated yield also improves as the alcohol gets heavier (64% for **A2**, 82% for **A3**%, and 85% for **A4**). The most likely reason for this is the lower volatility of heavier vinyl ethers, which do not readily evaporate during the drying of the product *in vacuo*.

Different benzylic alcohols were also tested. Products **A6**–**A10**, bearing electron‐withdrawing groups on the aromatic ring, were obtained in excellent conversions and mostly good isolated yields (67%–78%). Slightly better conversion of 87% was achieved with benzyl alcohol compared to the optimization experiments along with a very good isolated yield of the desired product of 86%. **A11** was formed in 76%, which suggests that the more electron‐rich aromatic ring of the substrate of this type is a disadvantage for the catalyst. Compound **A12**, bearing a very strong electron‐withdrawing group on the aromatic ring was only produced in 47%. 1,4‐Bis(hydroxymethyl)benzene bearing two −OH groups was used as the substrate to obtain **A13**. Seventy‐eight percent of −OH groups were converted to vinyl ether groups in this reaction. The isolated yield of 62% corresponds to pure **A13**. Furfuryl alcohol has afforded good conversion to **A14** with an average isolated yield of 53%.

Secondary alcohols were also tested as substrates. Transfer vinylation of cyclohexanol resulted in a very good conversion to **A15** of 80%; however, a poor isolated yield of 31% was achieved. All other secondary alcohols tested resulted in average conversions to the desired products (products **A16**–**A20**).

Product **A21** bearing ether and ester groups was formed in >95% and isolated in 67% yield. The pure product contained an intact ester group.

While catalyst **1** can perform transfer vinylation of different primary and secondary alcohols, the protocol has limitations. Tertiary alcohols and phenols do not undergo any transfer vinylation under the given conditions (**A22** and **A23**, more examples are shown in the Supporting Information 5). The presence of thiol, primary amine, and nitrile groups in the substrates also inhibits the reaction (**A24**–**A27**). An example shows (Supporting Information 3.7.1) that the addition of a primary amine to the experiment shown in Table 2, entry 2, reduces the yield of the product significantly. However, primary alcohols bearing BOC‐protected amine groups can undergo transfer vinylation, as shown in Supporting Information 5.

### 2.3 Recovery and Reutilization of Organic Reaction Materials

**Scheme 3:**
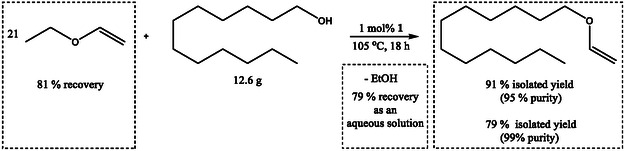
Transfer vinylation of dodecyl alcohol on a 12.6 g scale including separation of all organic components.

An experiment was performed to demonstrate the possibility of performing vinylation with minimal amounts of waste, which includes recovery or recycling of organic reaction components (**Scheme** **3**).

In order to recover ethyl vinyl ether, our first and most conservative approach was to do distillation of the crude reaction solution. In this case, the obtained vinyl ether was contaminated with 7% ethanol. As it is known that vinyl ethers can azeotrope with the parent alcohols,^[^ ^20^ ^]^ the formed ethanol was removed by extraction prior to distillation. Saturated brine was used for this purpose (5 × 35 g) to prevent the leaching of ethyl vinyl ether to the aqueous phase.

The remaining organic layer was subjected to distillation at 62 °C. This afforded mostly pure EtOVi with minor water/ethanol contamination (below 1%) which was removed by brine extraction (2 × 22 g), and the remaining ether was dried with sodium sulfate, affording very pure EtOVi (81.42 g, 81%) (Supporting Information 6.1).

The reaction shown in Table 2, entry 2, was performed with the isolated ethyl vinyl ether from this experiment, which afforded 84% yield of the benzyl vinyl ether, thus demonstrating that the vinylating agent can be recovered and reused.

The remaining organic layer was used for Kügelrorh distillation at 80 °C with Schlenk line vacuum affording the product in technical purity of 95% (13.1 g, 91%) (Supporting Information 6.2). In order to further purify the product, a filtration column with 90 g of silica was set up. The 95% pure product was introduced to the dry column and washed down with 150 mL hexane. The hexane was evaporated affording the pure product (11.527 g, overall yield of 79%) (Supporting Information 6.3).

All the brine solutions from the experiment were combined. Analyzing the sample by ^1^H NMR in D_2_O shows that ethanol is the only organic component present in the solution (Supporting Information 6.4). Distillation was performed to isolate the ethanol dissolved in brine. The concentration of EtOH was determined from ^1^H NMR in D_2_O by using sodium acetate as a mass standard. Two fractions were collected by distillation: 1) 3.617 g of 27% ethanol in water (0.99 g ethanol is present (Supporting Information 6.5.1), 32% of the expected amount) and 2) 4.6 g of 33% ethanol in water (1.47 g ethanol is present, 47% of the expected amount) (Supporting Information 6.5.2). The ethanol in the remaining brine solution from distillation was quantified by the same method (330 mg ethanol in 196 g brine solution, 11% of the expected amount) (Supporting Information 6.5.3). The total amount of ethanol present in all three solutions equals to 2.79 g (90% of the expected amount assuming full conversion of dodecyl alcohol to dodecyl vinyl ether), wherein 79% is present as a solution in pure water in 27%–33% concentrations. These aqueous solutions can be used for production of higher concentration ethanol.^[^ ^21^ ^]^ Recycling of Ru is done on a commercial scale by multiple companies from different types of waste.^[^ ^22^ ^]^ We did not investigate the recycling methods ourselves, but if the reaction was scaled to industrial levels, third‐party recycling would enable the recovery of Ru.

## 3 Conclusion

Herein, the first efficient Ru‐catalyzed transfer vinylation of alcohols was shown. Several ruthenium complexes are able to perform this reaction with bis(2‐methylallyl)(1,5‐cycloocta­diene)ruthenium(II) being able to catalyze it well in the absence of any base. The catalyst is very efficient in vinylating many primary alcohol groups. Transfer vinylation of secondary alcohols is also possible, although it proceeds with moderate yields. However, tertiary and phenolic –OH groups cannot be vinylated using this protocol. The reaction does not require any additional solvent, using the vinylating agent both as the reagent and solvent. Testing different alkyl vinyl ethers as vinylating agents revealed that the reaction proceeds more efficiently in the presence of vinyl ethers with shorter alkyl chains. It was also shown that the reaction can be performed with minimal loss of organic compounds which enables separation and reuse of ethyl vinyl ether and isolation of formed ethanol in solution with water.

## Supporting Information

The authors have cited additional references within the Supporting Information.^[23–29]^

## Conflict of Interest

The authors declare no conflict of interest.

## Supporting information

Supplementary Material

## Data Availability

The data that support the findings of this study are available in the Supporting Information of this article.
