# Supplementary material for: Ru(II)‐Catalyzed Transfer Vinylation of Alcohols
Source: ChemSusChem. 2025 Oct 17;18(24):e202501279. doi: 10.1002/cssc.202501279 (PMC12703438; doi:10.1002/cssc.202501279)
Supplement: Supplementary file 1 — Supplementary Material [file CSSC-18-e202501279-s001.pdf]

Ru(II)-Catalyzed Transfer Vinylation of Alcohols

S. Opačak, S. Tin

Supplementary information

Table of Contents

1. Experimental 4
    1.1. General procedures 4
        1.1.1. Catalyst screening and optimisation reactions 4
        1.1.2. Substrate screening reactions 4
        1.1.3. Isolation reactions 4
        1.1.4. NMR measurements 4
    1.2. Isolated compound data 4
2. Compound characterisation 7
    A2 7
    A3 8
    A4 9
    A5 10
    A6 11
    A7 12
    A8 13
    A9 14
    A10 15
    A11 16
    A13 17
    A14 19
    A15 20
    A16 21
    A18 22
    A21 23
3. Additional screening data 25
    3.1. Catalyst screening 25
    3.2. Phenantroline as a ligand 25
    3.3. Ligand screening with **1** 27
    3.4. Screening with Et_3N 28
    3.5. Purified and unpurified substrate comparison 30
    3.6. Comprehensive table of conversions with **1** and unpurified substrates 31
    3.7. Other screening data 32
        3.7.1. Primary amine addition 32
4. Conversion determination - example 33
5. Substrate scope – additional examples 34

6. Recovery and Reutilization of Organic Reaction Materials. ... 35
6.1. Recovered ethyl vinyl ether ^1H NMR spectra (300 MHz, C_6D_6) ... 36
6.2. Product in technical purity ^1H NMR spectra(300 MHz, C_6D_6) ... 36
6.3. Pure product ^1H NMR spectra (300 MHz, C_6D_6) ... 37
6.4. Brine ^1H NMR spectra (300 MHz, D_2O) ... 37
6.5. Recovered ethanol ^1H NMR spectra (300 MHz, D_2O) ... 38
6.5.1. Fraction 1 ... 38
6.5.2. Fraction 2 ... 38
6.5.3. Remaining brine solution ... 39
7. Vinyl acetate ... 40
7.1. General procedure ... 40
7.2. Catalyst and condition screening ... 41
References ... 48

1. Experimental

1.1. General procedures

1.1.1. Catalyst screening and optimisation reactions

Reactions were carried out in oven dried pressure tubes with Teflon stoppers. Reaction conditions: 1 mmol substrate, 2 ml butyl-vinyl ether, 5 mol% catalyst for 1st row transition metals / 1 mol% catalyst for other metals, 50 µL hexadecane (GC standard), 105 °C, argon, magnetic stirring. Tubes were loaded with catalysts and solids in a glovebox with Ar atmosphere, liquid reagents were added via syringes under Ar flow, no additional solvent was used. The charged tubes were put into a cold aluminium rack and heated up; reaction time was measured from the moment reactions reach 105 °C.
After 18 h the reaction vessels were cooled down, opened and filtered through a short (1 cm) silica plug which was then washed with 1 ml EtOAc. Analysis was performed by GC (machine: Agilent 8890, column: Agilent 19091J-433: 3 HP-5, method: flow 1.0 mL/min, temperature start[°C]/hold time[min]-temperature ramp[°C/min]-temperature after ramp [°C]: 35/5-15-110/6-25-200/8-25-320/15) and conversions were determined by comparing the measure analyte/mass standard ratios to theoretical ones.
Differences to the general method (condition variations) are documented when discussing the specific experiments.

1.1.2. Substrate screening reactions

Reactions were carried out in oven dried pressure tubes with Teflon stoppers. Reaction conditions: 1 mmol substrate, 2 ml ethyl-vinyl ether, 3.2 mg catalyst (bis(2-methylallyl)(1,5-cyclooctadiene)ruthenium(II), 1 mol%), 50 µL 1-Methylnaphthalene (0.37 mmol, mass standard), 105 °C, argon, magnetic stirring. Tubes were loaded with catalyst and solid substrates in a glovebox with Ar atmosphere, liquid reagents were added via syringes under Ar flow, no additional solvent was used. The charged tubes were put into a cold aluminium rack and heated up; reaction time was measured from the moment reactions reach 105 °C.
After 18 h the reaction vessels were cooled down, opened and filtered through a short (1 cm) silica plug which was then washed with 1 ml EtOAc, a sample for NMR was taken at this point. The reaction mixtures were then evaporated at 150-250 mBar on a rotavap for 10-20 minutes and another sample was taken for NMR. Conversions were determined by comparing the intensity of 1-Methylnaphthalene signal and the characteristic dd signal from the vinyl group (see ESI 4.), preferentially from the sample before evaporation, if visible, otherwise, the sample after evaporation was used.

1.1.3. Isolation reactions

Reactions were carried out in oven dried pressure tubes with Teflon stoppers. Reaction conditions (unless otherwise noted): 5 mmol substrate, 10 ml ethyl-vinyl ether, 105 °C, 16 mg catalyst (bis(2-methylallyl)(1,5-cyclooctadiene)ruthenium(II), 1 mol%), argon, magnetic stirring. Tubes were loaded with catalyst and solid substrates in a glovebox with Ar atmosphere, liquid reagents were added via syringes under Ar flow, no additional solvent was used. The charged tubes were put into a cold aluminium rack and heated up; reaction time was measured from the moment reactions reach 105 °C.
After 18 h the reaction vessels were cooled down, opened and filtered through a short (2-3 cm) silica plug which was then washed with 5 ml EtOAc. The reaction mixture was then evaporated directly onto silica and purified via automated flash chromatography (Hexane:EtOAc, starting with pure hexane up to 5% EtOAc over 15 minutes); products generally elute first from the mixture in the first couple of minutes. Certain compounds (**A4, A15**) were first distilled with Kugelrohr and then purified using a small silica column in a Pasteur pipette.

1.1.4. NMR measurements

NMR spectra were measured using AV-III 300 (Bruker) or AV-III HD 400 (Bruker) and labelled accordingly. All measurements were performed at 25 °C. NMR spectra labelled as $^{13}C\{^{1}H\}$ are performed as attached proton tests (APT).

1.2. Isolated compound data

A2[1] Substrate: 1-hexanol (628 µL, 5.00 mmol). Yield of product: 410 mg (64%), light brown oil.
^{1}H NMR (300 MHz, C_6D_6) δ 6.45 (ddt, *J* = 14.3, 6.8, 0.6 Hz, 1H), 4.19 (dd, *J* = 14.4, 1.7 Hz, 1H), 3.96 (dd, *J* = 6.8, 1.7 Hz, 1H), 3.43 (t, *J* = 6.5 Hz, 2H), 1.56 – 1.40 (m, 2H), 1.28 – 1.12 (m, 6H), 0.89 – 0.81 (m, 3H). $^{13}C\{^{1}H\}$

NMR (75 MHz, C_6D_6) δ 152.1, 85.7, 67.6, 31.5, 29.0, 25.7, 22.6, 13.8. GC-MS (ESI) m/z (%): 128 (3.49) $[M]^+$, calculated mass ($C_8H_{16}O$) = 128.1.

A3[2] Substrate: 1-octanol (785 µL, 5.00 mmol). Yield of product: 642 mg (82%), clear oil.
^{1}H NMR (300 MHz, C_6D_6) δ 6.46 (ddt, *J* = 14.3, 6.8, 0.6 Hz, 1H), 4.19 (dd, *J* = 14.3, 1.7 Hz, 1H), 3.97 (dd, *J* = 6.8, 1.7 Hz, 1H), 3.45 (t, *J* = 6.4 Hz, 2H), 1.58 – 1.43 (m, 2H), 1.29 – 1.15 (m, 10H), 0.91 – 0.86 (m, 3H). $^{13}C\{^1H\}$ NMR (75 MHz, C_6D_6) δ 152.1, 85.7, 67.6, 31.8, 29.3, 29.3, 29.1, 26.0, 22.7, 14.0. GC-MS (ESI) m/z (%): 156 (11.00) $[M]^+$, calculated mass ($C_{10}H_{20}O$) = 156.2.

A4[3] Substrate: 1-dodecanol (932 mg, 5 mmol). Yield of product: 899 mg (85%), clear oil.
^{1}H NMR (300 MHz, C_6D_6) δ 6.47 (dd, *J* = 14.4, 6.8 Hz, 1H), 4.20 (dd, *J* = 14.3, 1.7 Hz, 1H), 3.97 (dd, *J* = 6.8, 1.7 Hz, 1H), 3.46 (t, *J* = 6.4 Hz, 2H), 1.51 (dt, *J* = 8.1, 6.4 Hz, 2H), 1.26 (d, *J* = 14.4 Hz, 18H), 0.94 – 0.86 (m, 3H). $^{13}C\{^1H\}$ NMR (75 MHz, C_6D_6) δ 152.1, 85.7, 67.6, 29.8, 29.7, 29.7, 29.7, 29.5, 29.4, 29.1, 22.8, 14.0. GC-MS (ESI) m/z (%): 212 (3.27) $[M]^+$, calculated mass ($C_{14}H_{28}O$) = 212.2.

A5[4] Substrate: Benzyl alcohol (500 µL, 5.00 mmol). Yield of product: 576 mg (86%), light brown oil.
^{1}H NMR (300 MHz, C_6D_6) δ 7.15 – 7.02 (m, 5H), 6.43 (ddd, *J* = 14.1, 6.8, 0.6 Hz, 1H), 4.44 (s, 2H), 4.22 (dd, *J* = 14.3, 2.0 Hz, 1H), 3.98 (dd, *J* = 6.8, 2.0 Hz, 1H). $^{13}C\{^1H\}$ NMR (101 MHz, C_6D_6) δ 151.6, 137.2, 128.2, 127.6, 127.3, 87.0, 69.7. GC-MS (ESI) m/z (%): 134 (1.06) $[M]^+$, calculated mass ($C_9H_{10}O$) = 134.1.

A6[5] Substrate: 4-fluorobenzyl alcohol (545 µL, 5 mmol). Yield of product: 590 mg (78%), light brown oil.
^{1}H NMR (300 MHz, C_6D_6) δ 6.99 – 6.84 (m, 2H), 6.73 (t, *J* = 8.7 Hz, 2H), 6.39 (ddt, *J* = 14.3, 6.8, 0.5 Hz, 1H), 4.27 (s, 2H), 4.17 (dd, *J* = 14.3, 2.1 Hz, 1H), 3.97 (dd, *J* = 6.8, 2.1 Hz, 1H). $^{13}C\{^1H\}$ NMR (75 MHz, C_6D_6) δ 164.1, 160.8, 151.5, 129.1 (d, *J* = 8.2 Hz), 115.0 (d, *J* = 21.5 Hz), 87.0, 68.9. GC-MS (ESI) m/z (%): 152 (1.27) $[M]^+$, calculated mass (C_9H_9FO) = 152.1.

A7[4] Substrate: 4-Chlorobenzyl alcohol (715 mg, 5 mmol). Yield of product: 616 mg (73%), light yellow oil.
^{1}H NMR (400 MHz, C_6D_6) δ 7.09 – 7.01 (m, 2H), 6.86 – 6.77 (m, 2H), 6.36 (dd, *J* = 14.3, 6.8 Hz, 1H), 4.23 (s, 2H), 4.15 (dd, *J* = 14.3, 2.1 Hz, 1H), 3.97 (dd, *J* = 6.8, 2.1 Hz, 1H). $^{13}C\{^1H\}$ NMR (75 MHz, C_6D_6) δ 151.4, 135.5, 133.4, 128.6, 128.4, 87.1, 68.7. GC-MS (ESI) m/z (%): 168 (2.54) $[M]^+$, calculated mass (C_9H_9ClO) = 168.0.

A8[4] Substrate: 4-(Trifluoromethyl)benzyl alcohol (685 µL, 5 mmol). Yield of product: 729 mg (72%), yellow oil.
^{1}H NMR (300 MHz, C_6D_6) δ 7.32 – 7.23 (m, 2H), 6.96 – 6.86 (m, 2H), 6.35 (ddt, *J* = 14.3, 6.8, 0.5 Hz, 1H), 4.24 (s, 2H), 4.13 (dd, *J* = 14.3, 2.2 Hz, 1H), 3.97 (dd, *J* = 6.8, 2.2 Hz, 1H). $^{13}C\{^1H\}$ NMR (75 MHz, C_6D_6) δ 151.2, 141.0, 127.1, 125.1 (q, *J* = 3.8 Hz), 122.7, 87.3, 68.5. GC-MS (ESI) m/z (%): 202 (1.57) $[M]^+$, calculated mass ($C_{10}H_9F_3O$) = 202.1.

A9[6] Substrate: 3,5-dichlorobenzyl alcohol (885 mg, 5 mmol). Yield of product: 686 mg (68%), clear oil.
^{1}H NMR (400 MHz, C_6D_6) δ 7.03 – 6.97 (m, 1H), 6.89 – 6.82 (m, 2H), 6.24 (dd, *J* = 14.3, 6.9 Hz, 1H), 4.04 (dd, *J* = 14.3, 2.3 Hz, 1H), 4.00 (s, 2H), 3.93 (dd, *J* = 6.8, 2.3 Hz, 1H). $^{13}C\{^1H\}$ NMR (75 MHz, C_6D_6) δ 151.0, 140.5, 134.9, 127.6, 125.3, 87.5, 67.7. GC-MS (ESI) m/z (%): 204 (52.99) $[M+2H]^+$, calculated mass ($C_9H_8Cl_2O$) = 202.0.

A10[6] Substrate: 2,6-dichlorobenzyl alcohol (885 mg, 5 mmol). Yield of product: 685 mg (67%), light yellow oil.
^{1}H NMR (300 MHz, C_6D_6) δ 6.82 (d, *J* = 8.1 Hz, 2H), 6.49 (ddt, *J* = 14.2, 6.8, 0.6 Hz, 1H), 6.39 (t, *J* = 8.1 Hz, 1H), 4.76 (s, 2H), 4.28 (dd, *J* = 14.2, 2.2 Hz, 1H), 3.99 (dd, *J* = 6.8, 2.2 Hz, 1H). $^{13}C\{^1H\}$ NMR (75 MHz, C_6D_6) δ 151.7, 136.8, 128.0, 86.8, 64.5. GC-MS (ESI) m/z (%): 202 (1.57) $[M]^+$, calculated mass ($C_9H_8Cl_2O$) = 202.0.

A11[4] Substrate: Anisyl alcohol (620 µL, 5 mmol). Yield of product: 481 mg (57%), brown oil.
^{1}H NMR (300 MHz, C_6D_6) δ 7.13 – 7.07 (m, 2H), 6.81 – 6.68 (m, 2H), 6.48 (ddt, *J* = 14.3, 6.8, 0.5 Hz, 1H), 4.44 (s, 2H), 4.26 (dd, *J* = 14.3, 1.9 Hz, 1H), 4.00 (dd, *J* = 6.8, 1.9 Hz, 1H), 3.26 (s, 3H). $^{13}C\{^1H\}$ NMR (75 MHz, C_6D_6) δ 159.6, 151.7, 129.1, 113.8, 86.8, 69.6, 54.4. GC-MS (ESI) m/z (%): 164 (1.18) $[M]^+$, calculated mass ($C_{10}H_{12}O_2$) = 164.1.

A13[7] Substrate: Benzene dimethanol (349 mg, 2.50 mmol). Yield of product: 296 mg (62%), slightly yellow crystals.
^{1}H NMR (300 MHz, C_6D_6) δ 7.12 (s, 4H), 6.50 – 6.37 (m, 2H), 4.42 (s, 4H), 4.22 (dd, *J* = 14.3, 2.0 Hz, 2H), 3.98 (dd, *J* = 6.8, 2.0 Hz, 2H). $^{13}C\{^1H\}$ NMR (75 MHz, C_6D_6) δ 151.6, 136.6, 87.0, 69.4. ATR-IR [cm^{-1}]: 2939,

2883, 1615, 1539, 1475, 1420, 1403, 1376, 1316, 1177, 1109, 1073, 1021, 979, 958, 821, 786, 712, 580, 549. GC-MS (ESI) m/z (%): 147 (93.77) $[M-C_2H_3O]^+$, calculated mass ($C_{10}H_{11}O$) = 147.1.

A14[8] Substrate: Furfuryl alcohol (435 µL, 5.00 mmol). Yield of product: 329 mg (53%), brown oil.
^{1}H NMR (300 MHz, C_6D_6) δ 7.02 (dd, *J* = 1.8, 0.9 Hz, 1H), 6.35 (ddt, *J* = 14.2, 6.8, 0.5 Hz, 1H), 6.05 – 5.94 (m, 2H), 4.35 (s, 2H), 4.20 (dd, *J* = 14.3, 2.1 Hz, 1H), 3.94 (dd, *J* = 6.8, 2.1 Hz, 1H). ^{13}C{^{1}H} NMR (101 MHz, C_6D_6) δ 151.2, 150.6, 142.7, 110.2, 109.4, 87.1, 62.1. GC-MS (ESI) m/z (%): 124 (3.46) $[M]^+$, calculated mass ($C_7H_8O_2$) = 124.1.

A15[3] Substrate: Cyclohexanole (500 mg, 5 mmol). Yield of product: 194 mg (31%), clear oil.
^{1}H NMR (300 MHz, C_6D_6) δ 6.24 (dd, *J* = 14.2, 6.6 Hz, 1H), 4.43 (dd, *J* = 14.1, 1.2 Hz, 1H), 4.02 (dd, *J* = 6.6, 1.3 Hz, 1H), 3.49 (tt, *J* = 8.8, 3.8 Hz, 1H), 1.71 (s, 2H), 1.63 – 1.47 (m, 2H), 1.46 – 1.17 (m, 3H), 1.15 – 0.94 (m, 3H). ^{13}C{^{1}H} NMR(75 MHz, C_6D_6) δ 150.7, 87.7, 76.7, 31.8, 25.4, 23.4. GC-MS (ESI) m/z (%): 126 (15.20) $[M]^+$, calculated mass ($C_8H_{14}O$) = 126.1.

A16[1] Substrate: 2-hexanol (630 µL, 5 mmol). Yield of product: 286 mg (46%), light orange oil.
^{1}H NMR (300 MHz, C_6D_6) δ 6.23 (ddd, *J* = 14.1, 6.6, 0.5 Hz, 1H), 4.38 (dd, *J* = 14.2, 1.3 Hz, 1H), 4.00 (dd, *J* = 6.6, 1.3 Hz, 1H), 3.68 – 3.52 (m, 1H), 1.57 – 1.44 (m, 1H), 1.27 – 1.20 (m, 5H), 1.03 (d, *J* = 6.1 Hz, 3H), 0.86 – 0.80 (m, 3H). ^{13}C{^{1}H} NMR(101 MHz, C_6D_6) δ 151.1, 87.4, 75.3, 36.1, 31.6, 27.5, 22.7, 22.6, 13.9. GC-MS (ESI) m/z (%): 128 (4.13) $[M]^+$, calculated mass ($C_8H_{16}O$) = 128.1.

A18[2] Substrate: 1-phenylethanol (605 µL, 5 mmol). Yield of product: 287 mg (39%), yellow oil.
^{1}H NMR (400 MHz, C_6D_6) δ 7.15 – 7.09 (m, 4H), 7.07 – 6.99 (m, 1H), 6.22 (dd, *J* = 14.2, 6.7 Hz, 1H), 4.59 (q, *J* = 6.5 Hz, 1H), 4.35 (dd, *J* = 14.2, 1.6 Hz, 1H), 3.95 (dd, *J* = 6.7, 1.5 Hz, 1H), 1.32 (d, *J* = 6.5 Hz, 3H). ^{13}C{^{1}H} NMR (75 MHz, C_6D_6) δ 150.6, 143.2, 128.4, 127.3, 125.7, 88.9, 77.1, 23.5. GC-MS (ESI) m/z (%): 148 (0.92) $[M]^+$, calculated mass ($C_{10}H_{12}O$) = 148.1.

A21 Substrate: tert-Butyl 12-hydroxy-4,7,10-trioxadodecanoate (1325 µL, 5 mmol). Yield of product: 1026 mg (67%), light brown oil.
^{1}H NMR (400 MHz, C_6D_6) δ 6.41 (dd, *J* = 14.3, 6.8 Hz, 1H), 4.14 (dd, *J* = 14.3, 1.9 Hz, 1H), 3.92 (dd, *J* = 6.8, 1.9 Hz, 1H), 3.60 – 3.52 (m, 4H), 3.44 – 3.39 (m, 10H), 2.36 (t, *J* = 6.4 Hz, 2H), 1.37 (s, 9H). ^{13}C{^{1}H} NMR (75 MHz, C_6D_6) δ 170.2, 151.9, 86.1, 79.4, 70.7, 70.6, 70.6, 70.4, 69.5, 67.4, 66.8, 36.3, 27.8. ATR-IR [cm^{-1}]: 2871, 1127, 1617, 1456, 1392, 1366, 1321, 1251, 1201, 1107, 981, 846, 817. Elemental analysis – calculated C: 59.19 %; H: 9.27 %, found C: 59.51 %; H: 9.51 %. GC-MS (ESI) m/z (%): 304 (0.06) $[M]^+$, 247 (0.53) $[M\text{-tBut}]^+$, calculated mass ($C_{15}H_{28}O_6$) = 304.2.

2. Compound characterisation

A2

^{1}H NMR (300 MHz, C_6D_6)

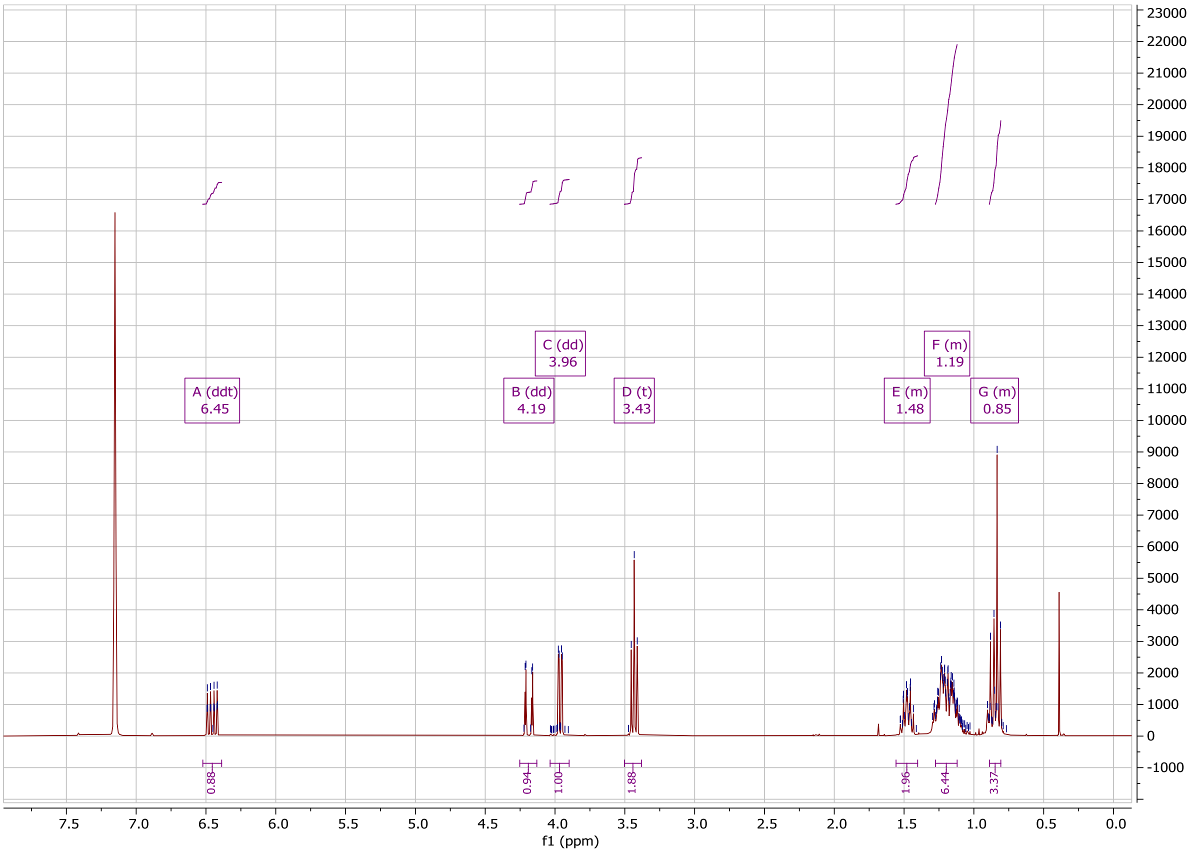

$^{13}C\{^{1}H\}$ NMR(75 MHz, C_6D_6)

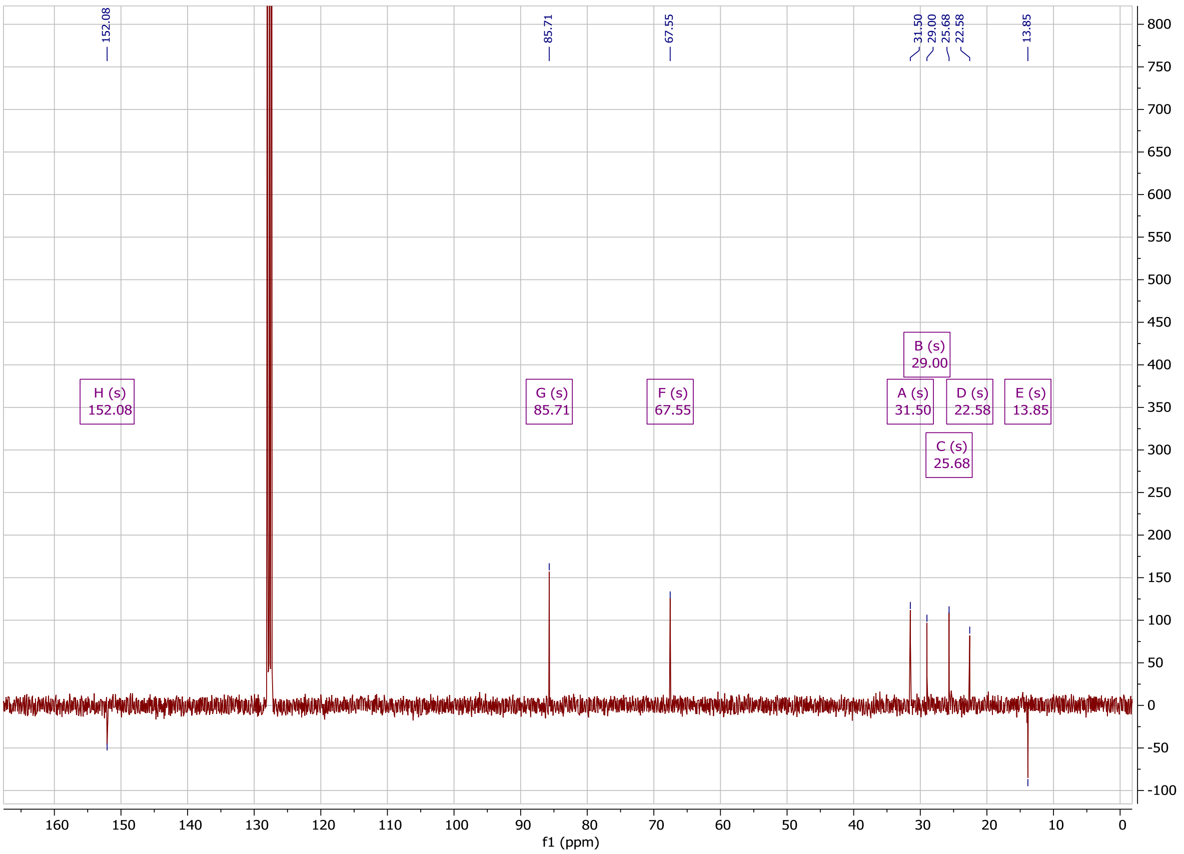

A3

^{1}H NMR (300 MHz, C_6D_6)

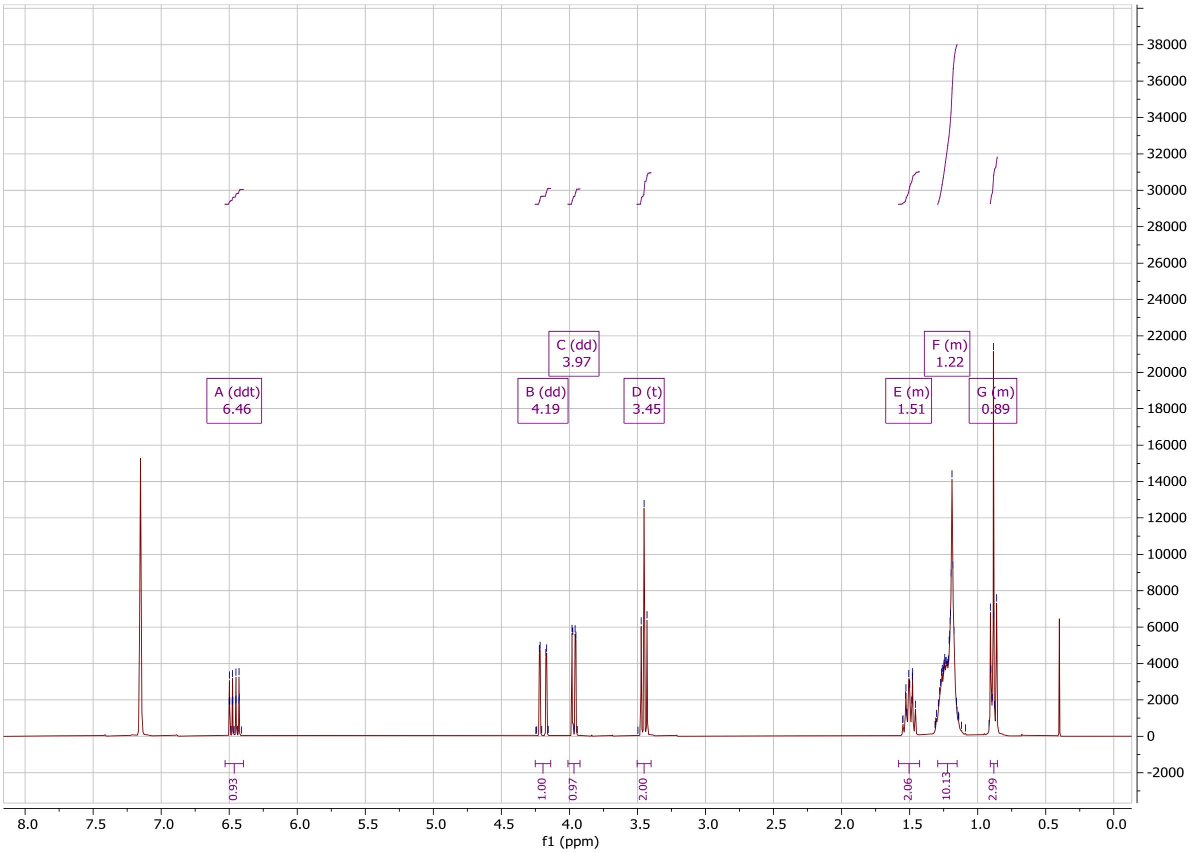

$^{13}C\{^{1}H\}$ NMR(75 MHz, C_6D_6)

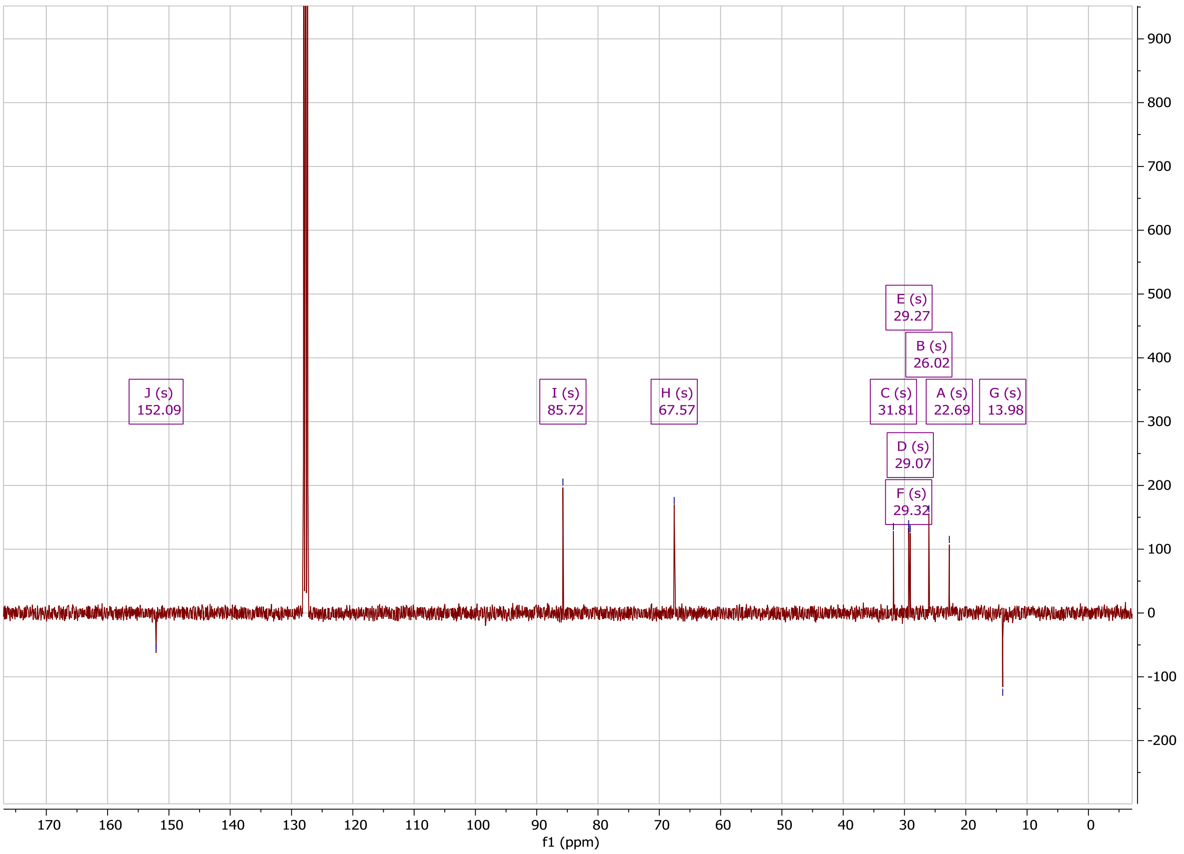

A4

^{1}H NMR (300 MHz, C_6D_6)

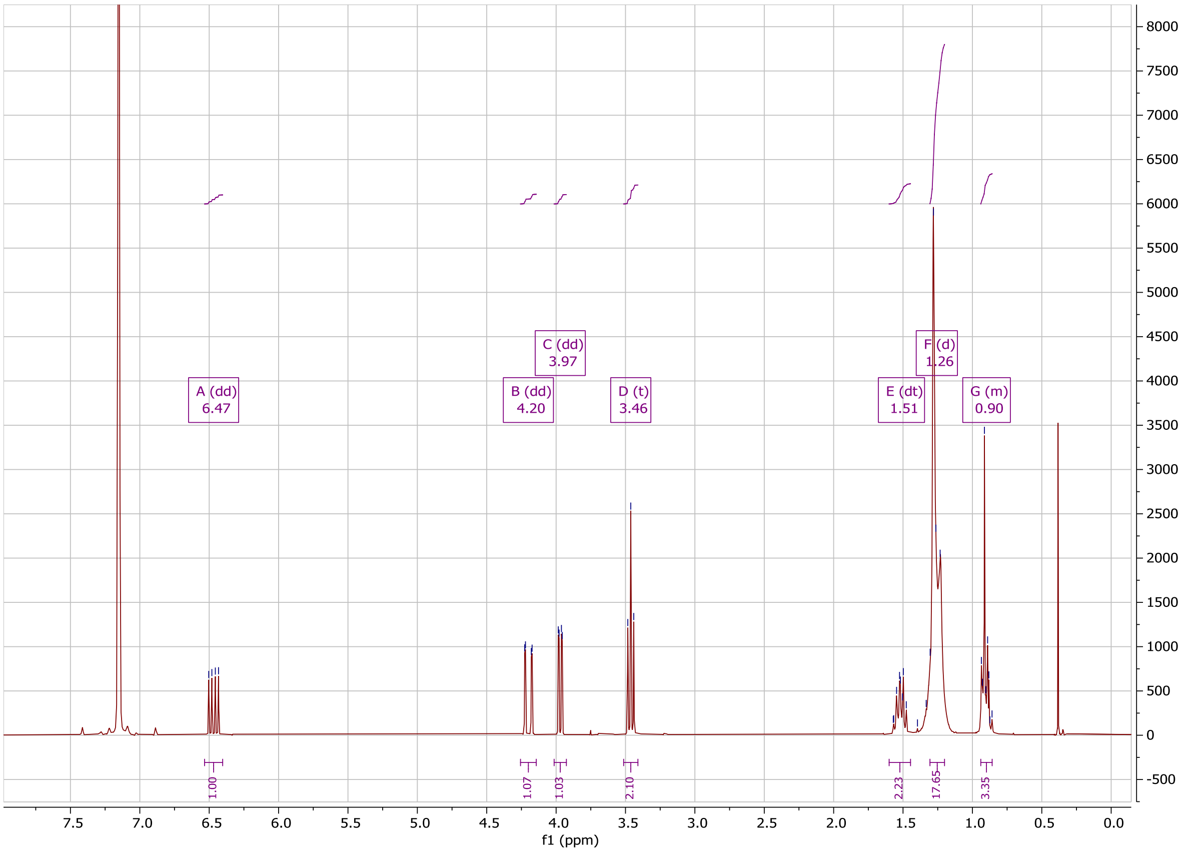

$^{13}C\{^{1}H\}$ NMR(75 MHz, C_6D_6)

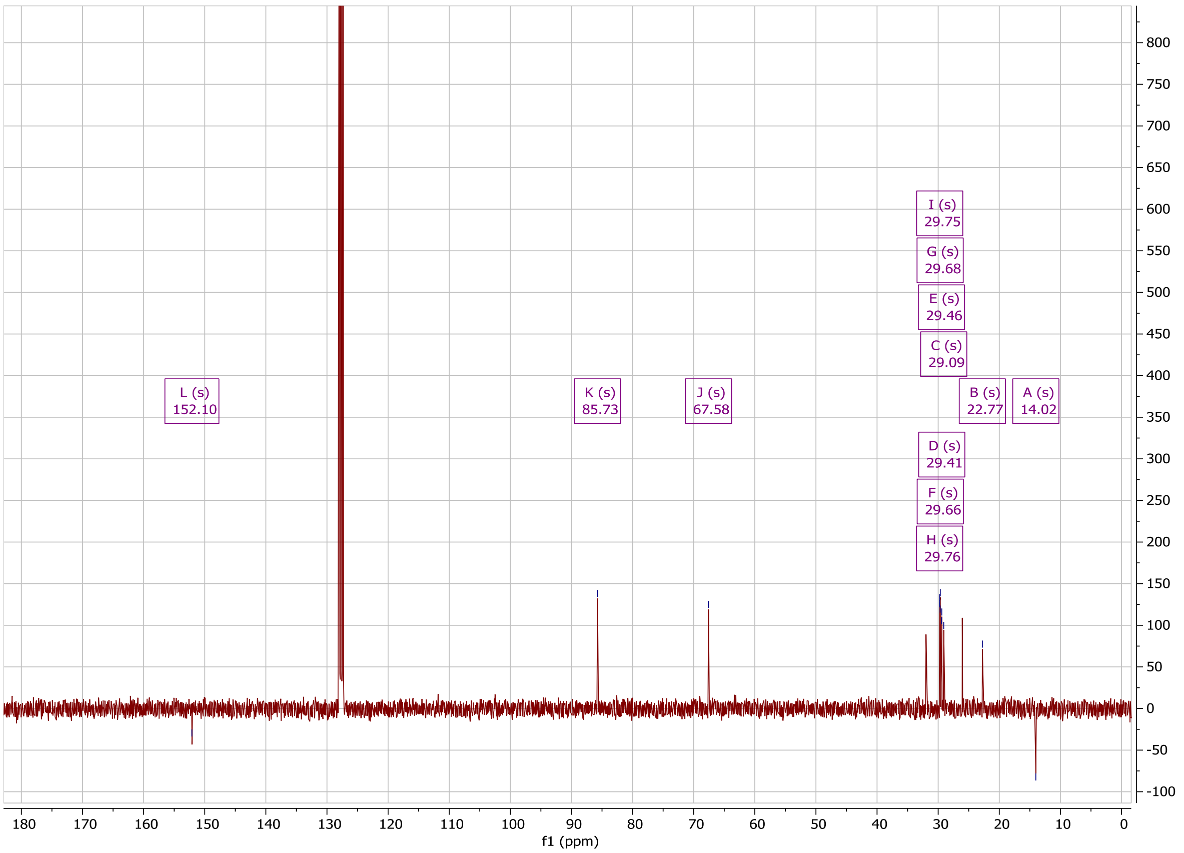

A5

^{1}H NMR (300 MHz, C_6D_6)

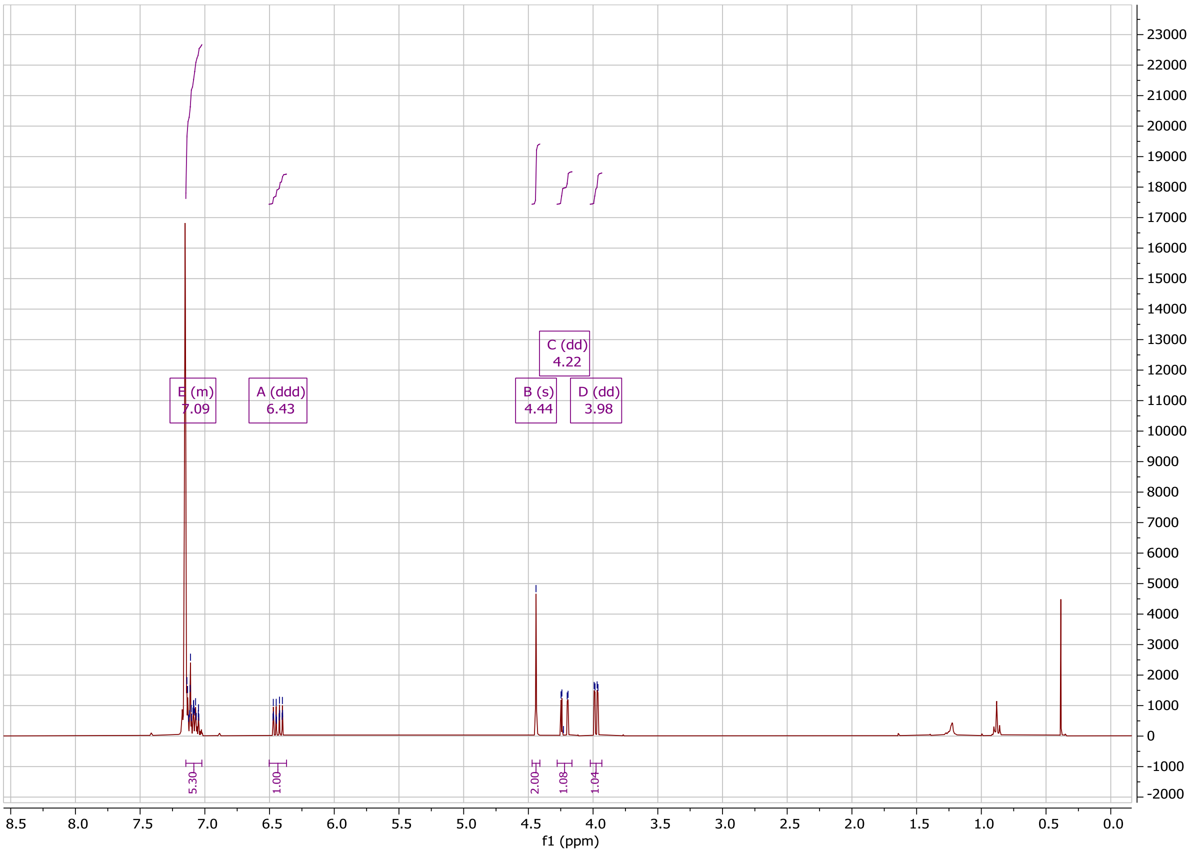

$^{13}C\{^{1}H\}$ NMR(75 MHz, C_6D_6)

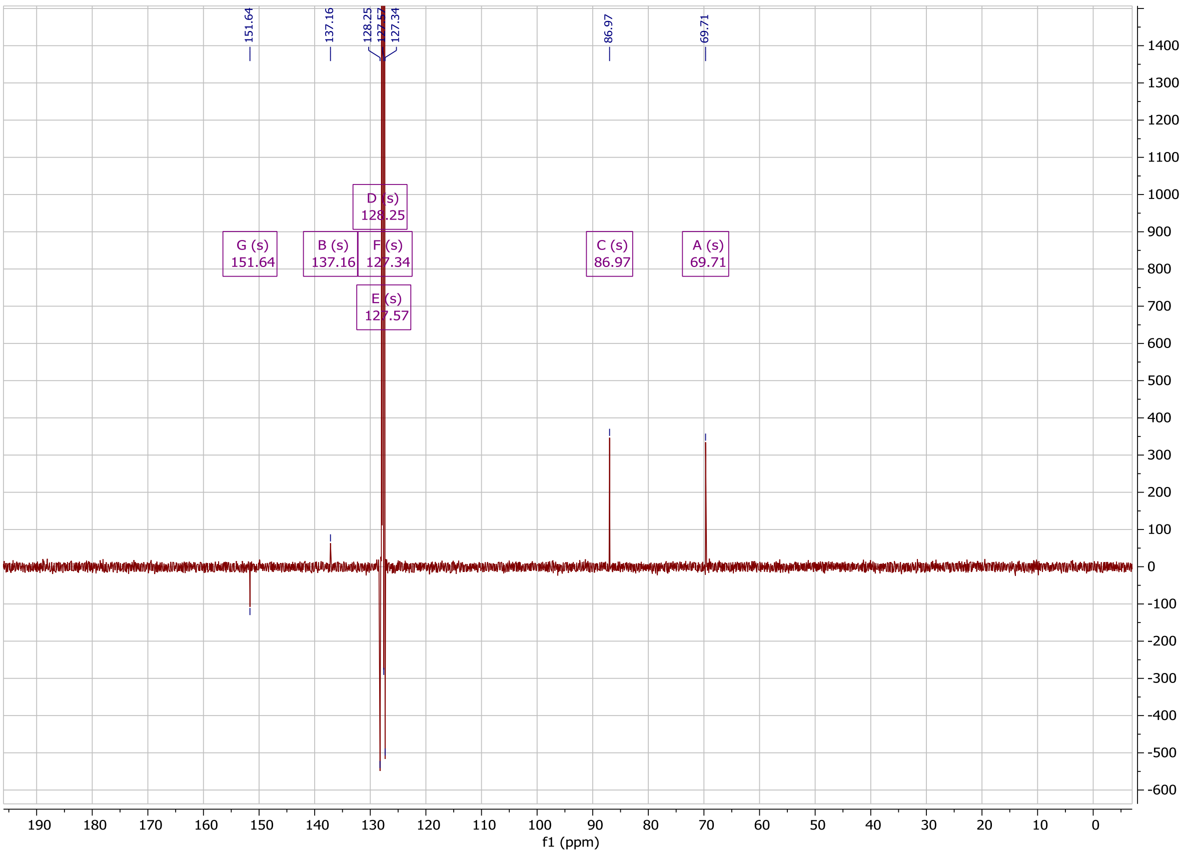

A6

^{1}H NMR (300 MHz, C_6D_6)

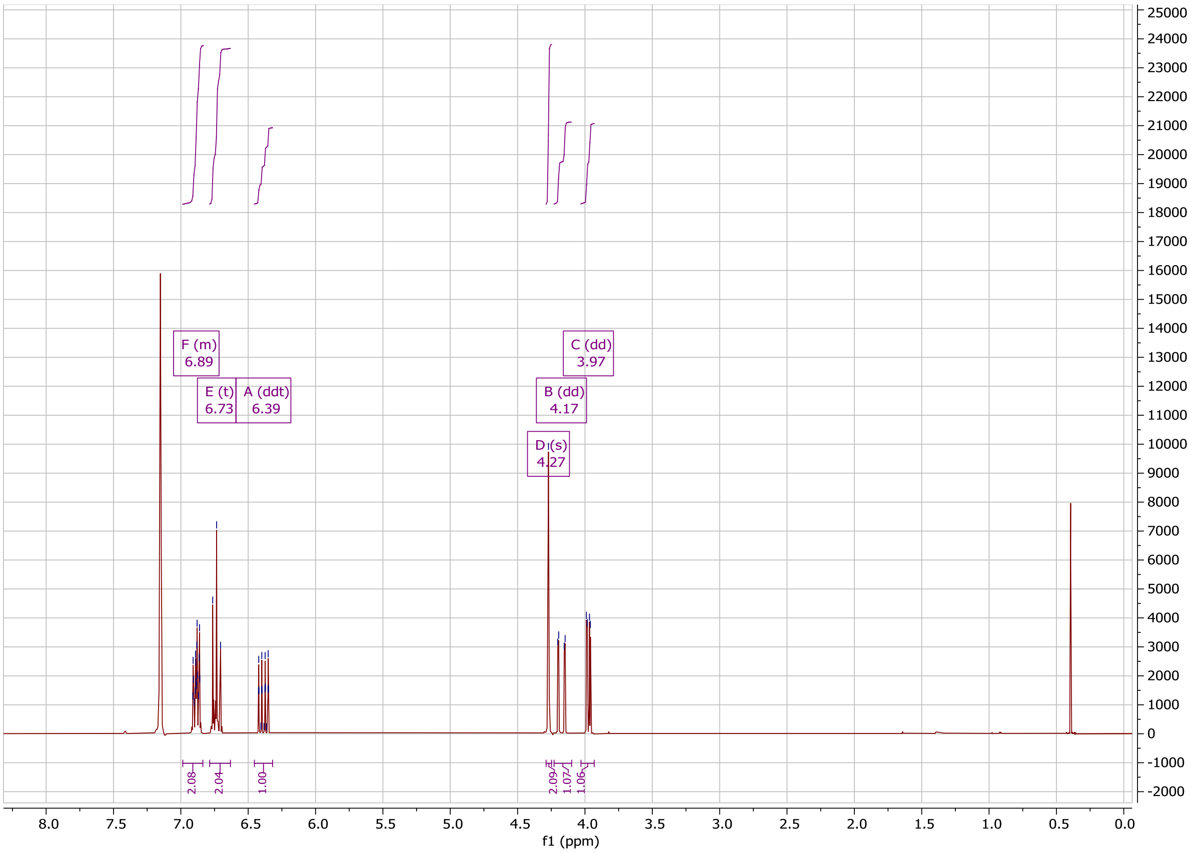

$^{13}C\{^{1}H\}$ NMR(75 MHz, C_6D_6)

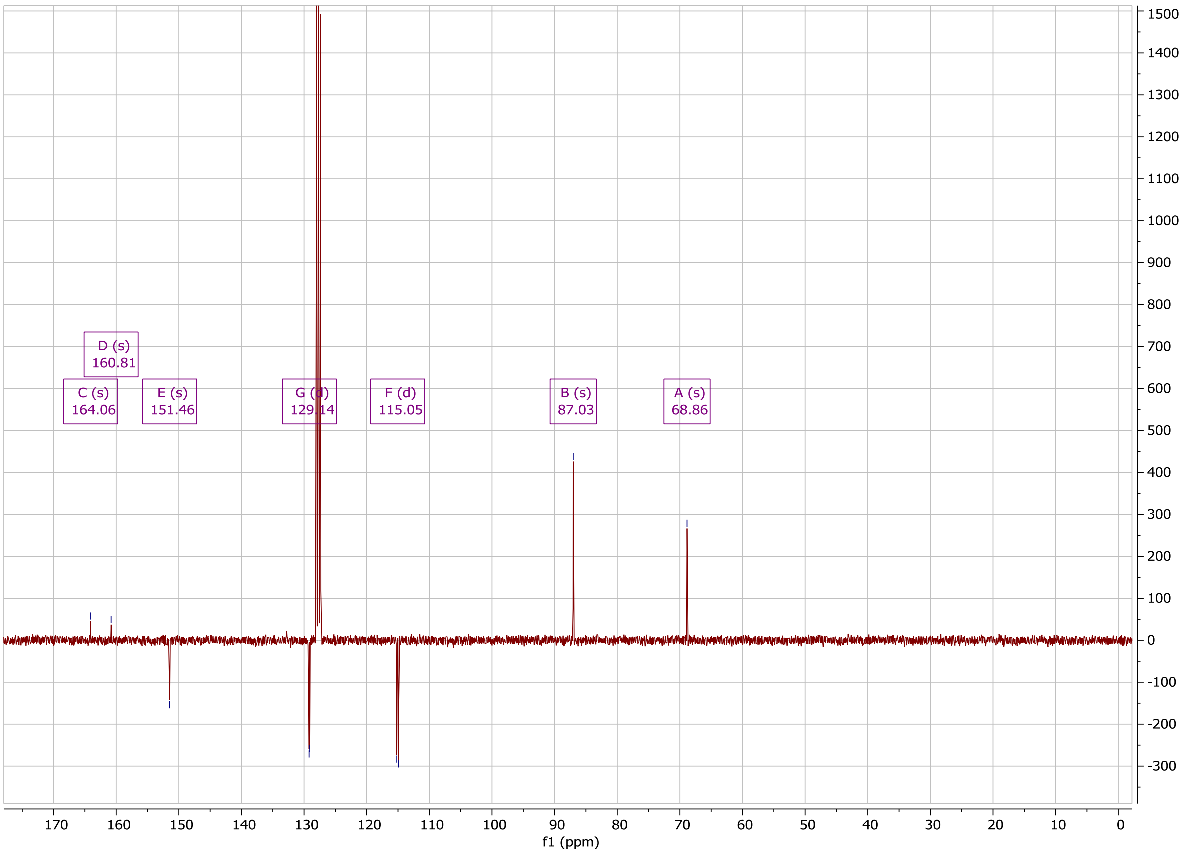

A7

^{1}H NMR (300 MHz, C_6D_6)

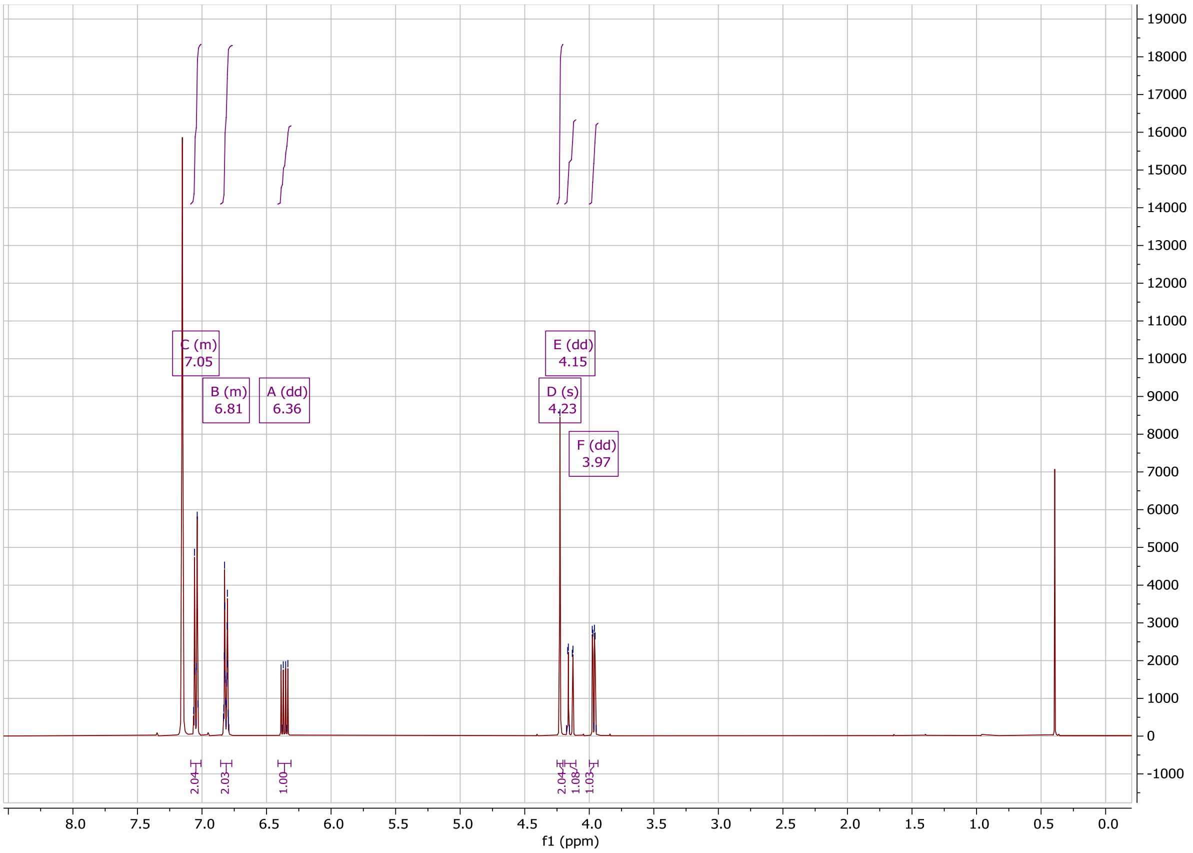

$^{13}C\{^1H\}$ NMR(75 MHz, C_6D_6)

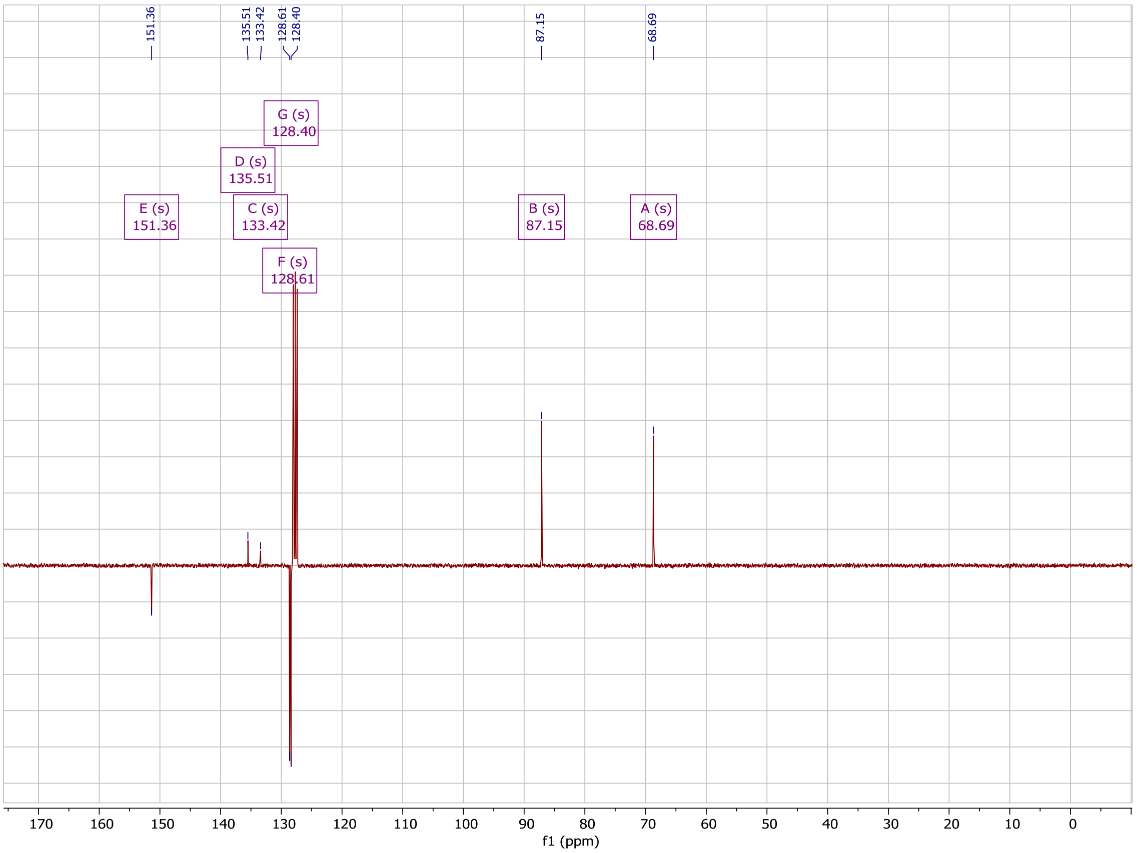

A8

^1H NMR (300 MHz, C_6D_6)

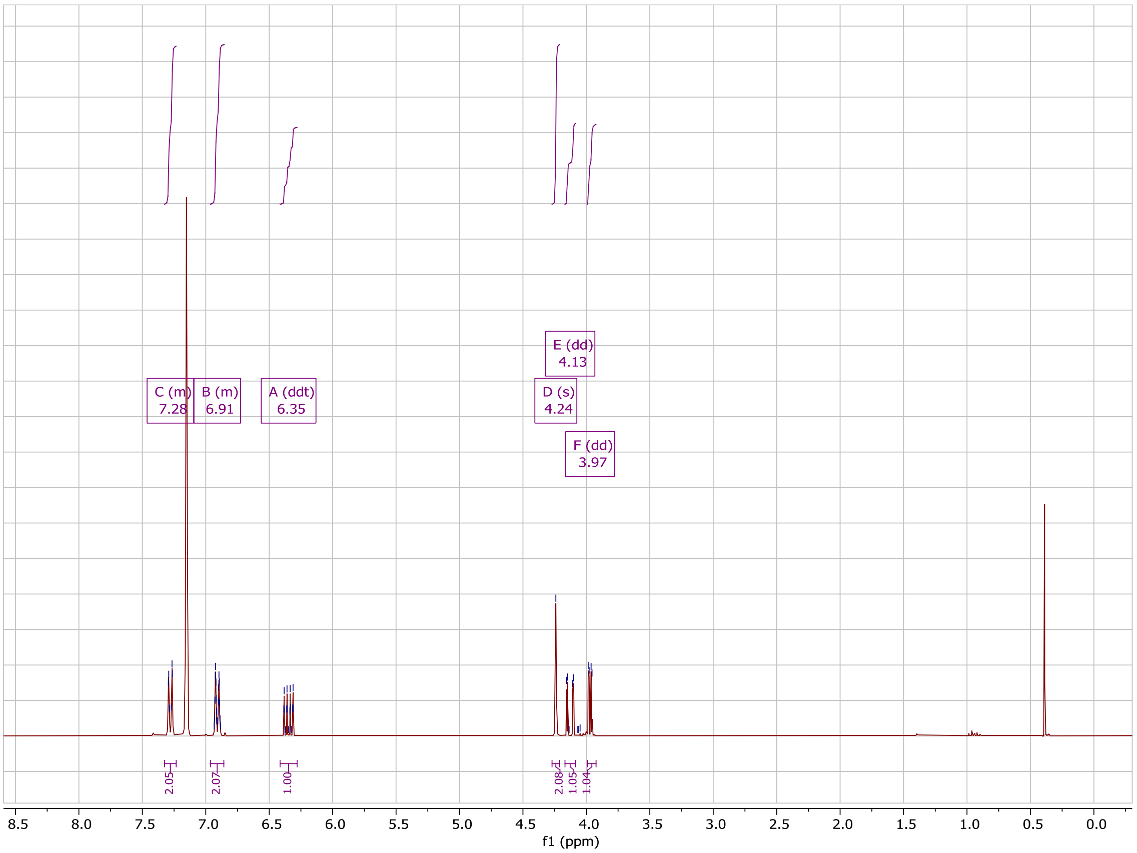

$^{13}C\{^1H\}$ NMR(75 MHz, C_6D_6)

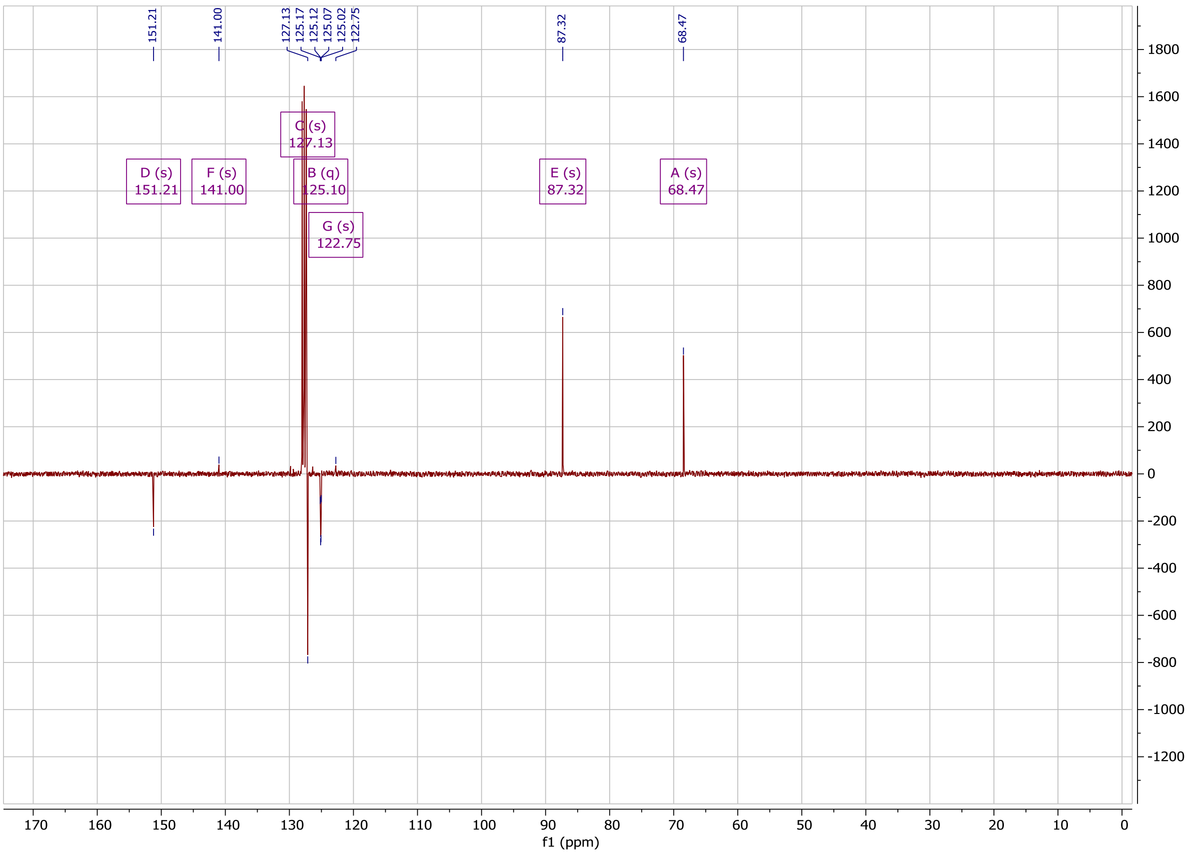

A9

^1H NMR (300 MHz, C_6D_6)

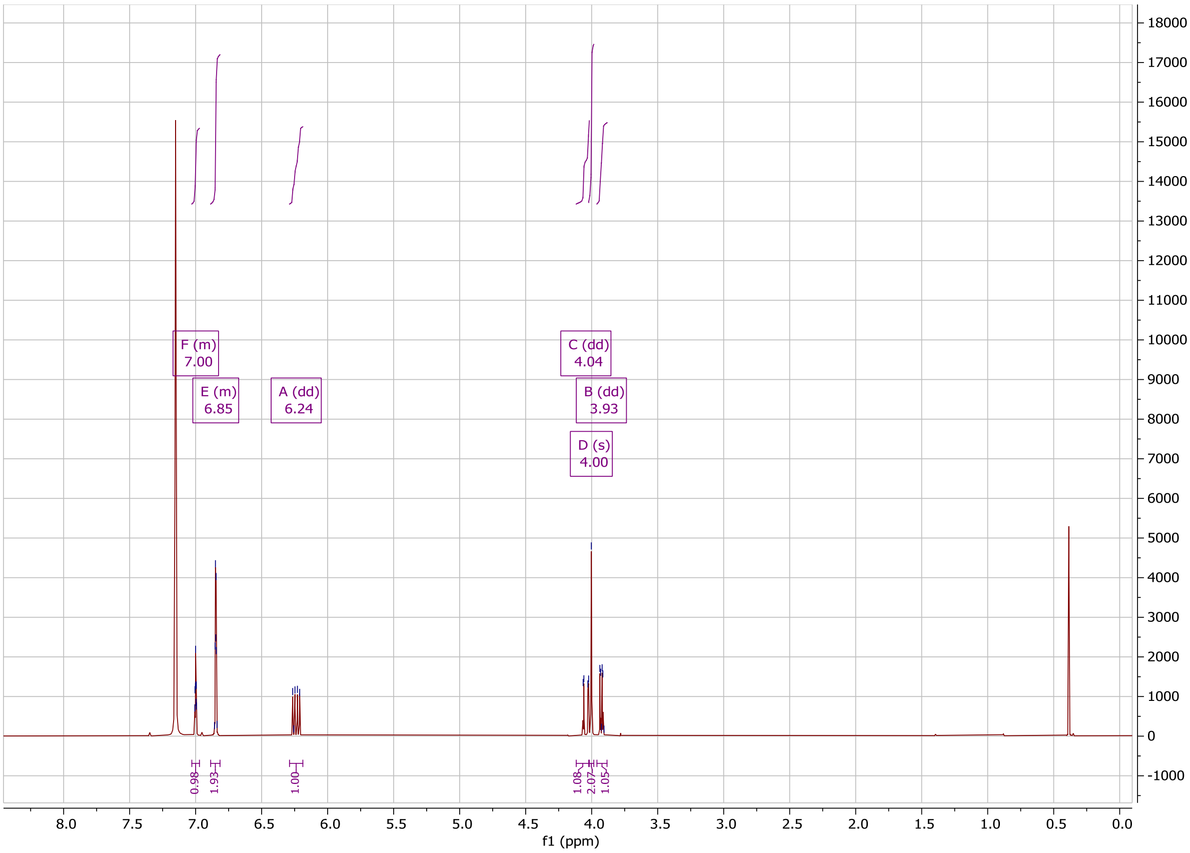

$^{13}C\{^{1}H\}$ NMR(75 MHz, C_6D_6)

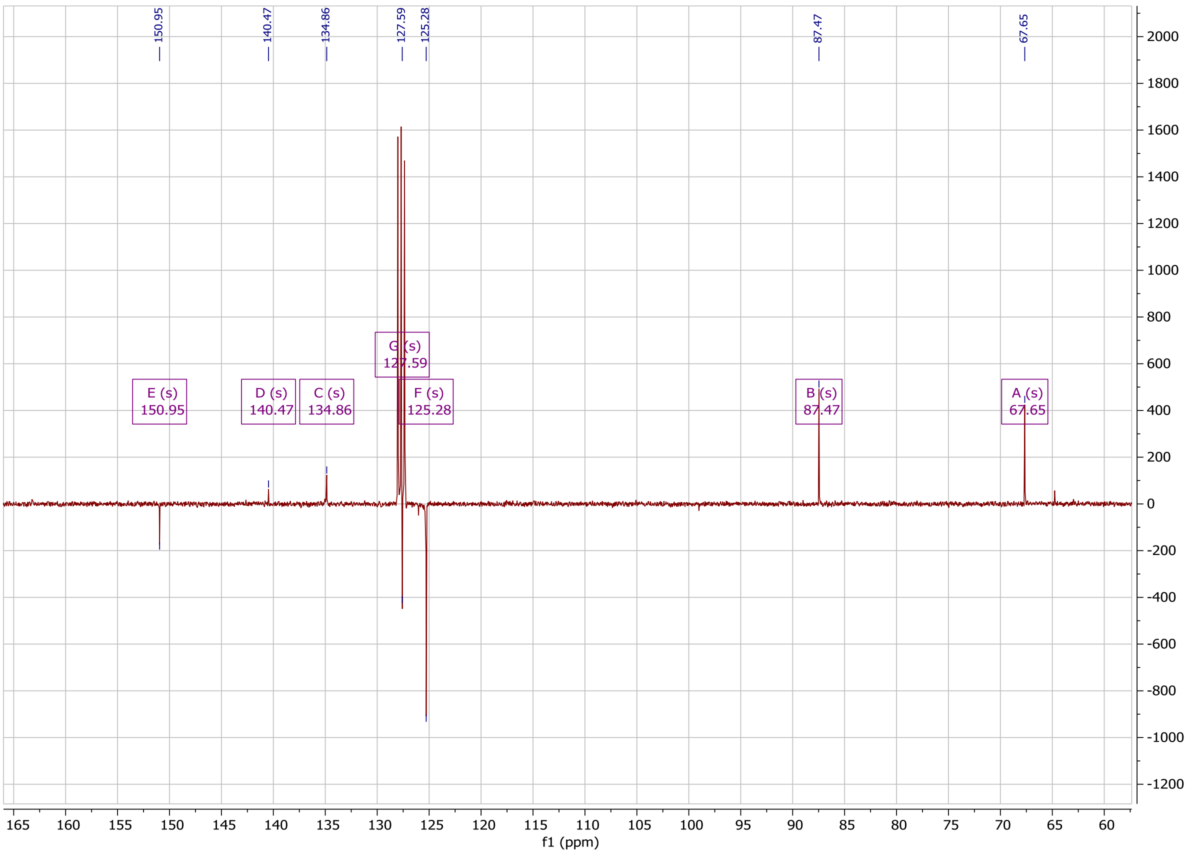

A10

^{1}H NMR (300 MHz, C_6D_6)

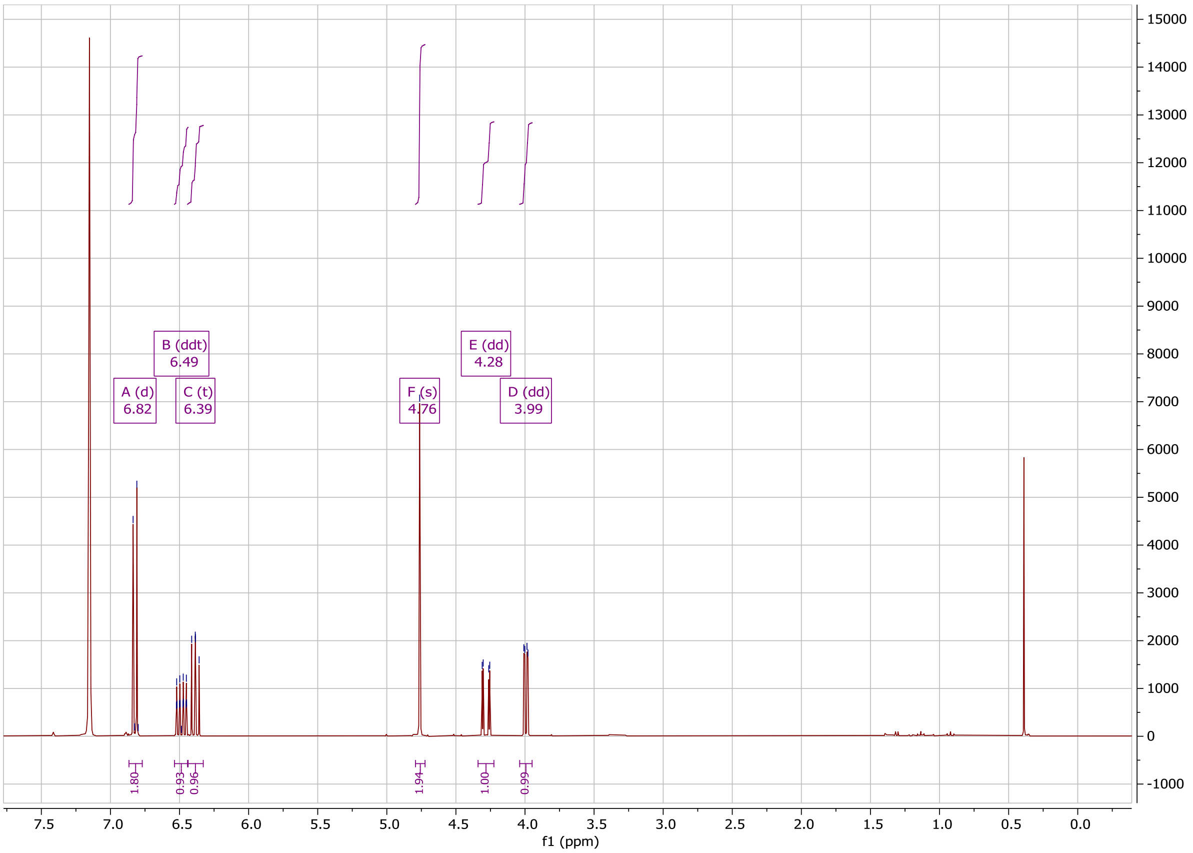

$^{13}C\{^1H\}$ NMR(75 MHz, C_6D_6)

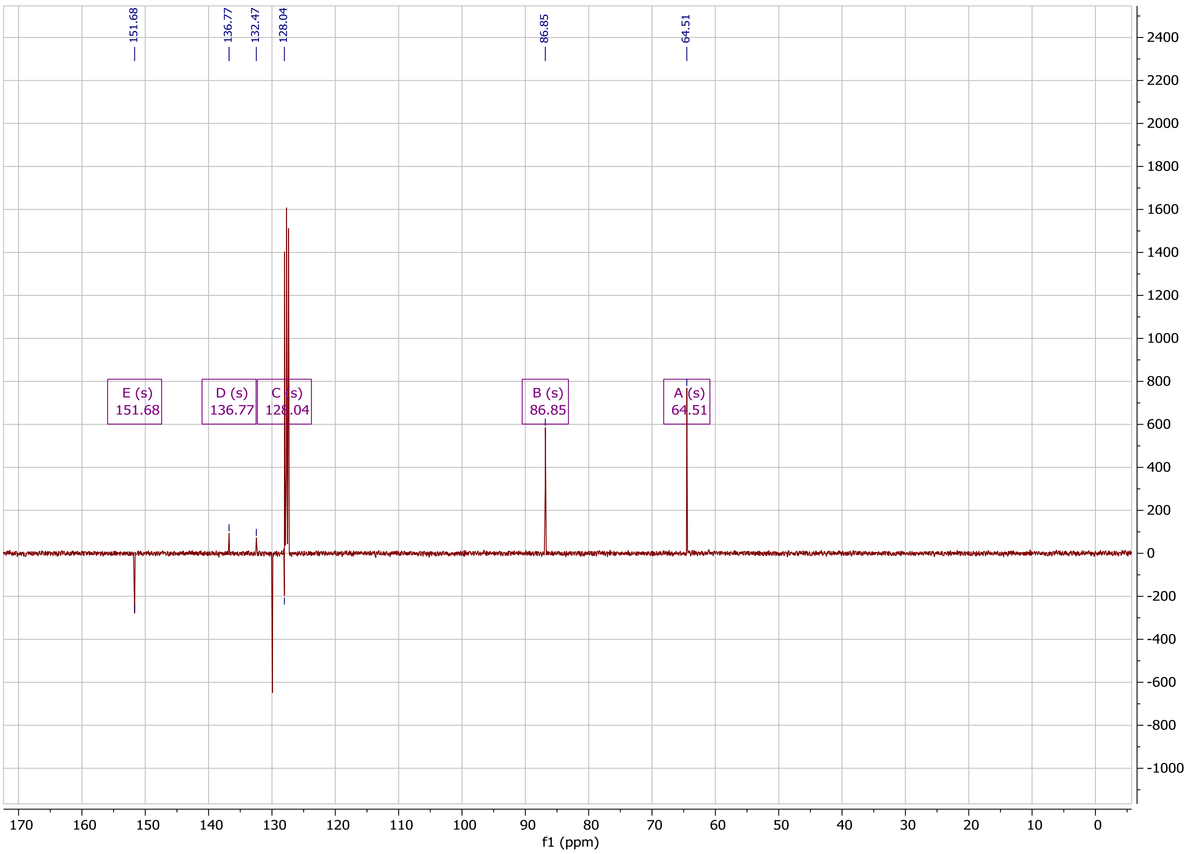

A11

^1H NMR (300 MHz, C_6D_6)

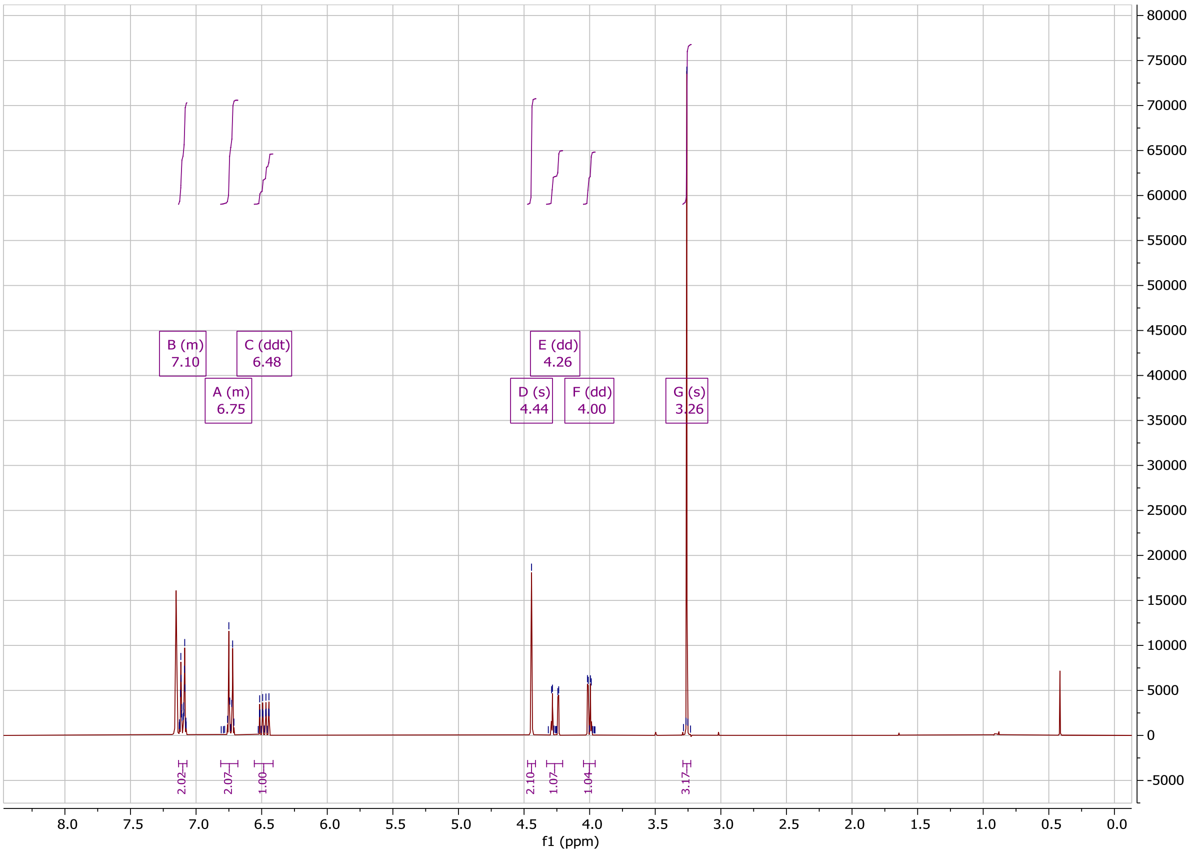

$^{13}C\{^{1}H\}$ NMR(75 MHz, C_6D_6)

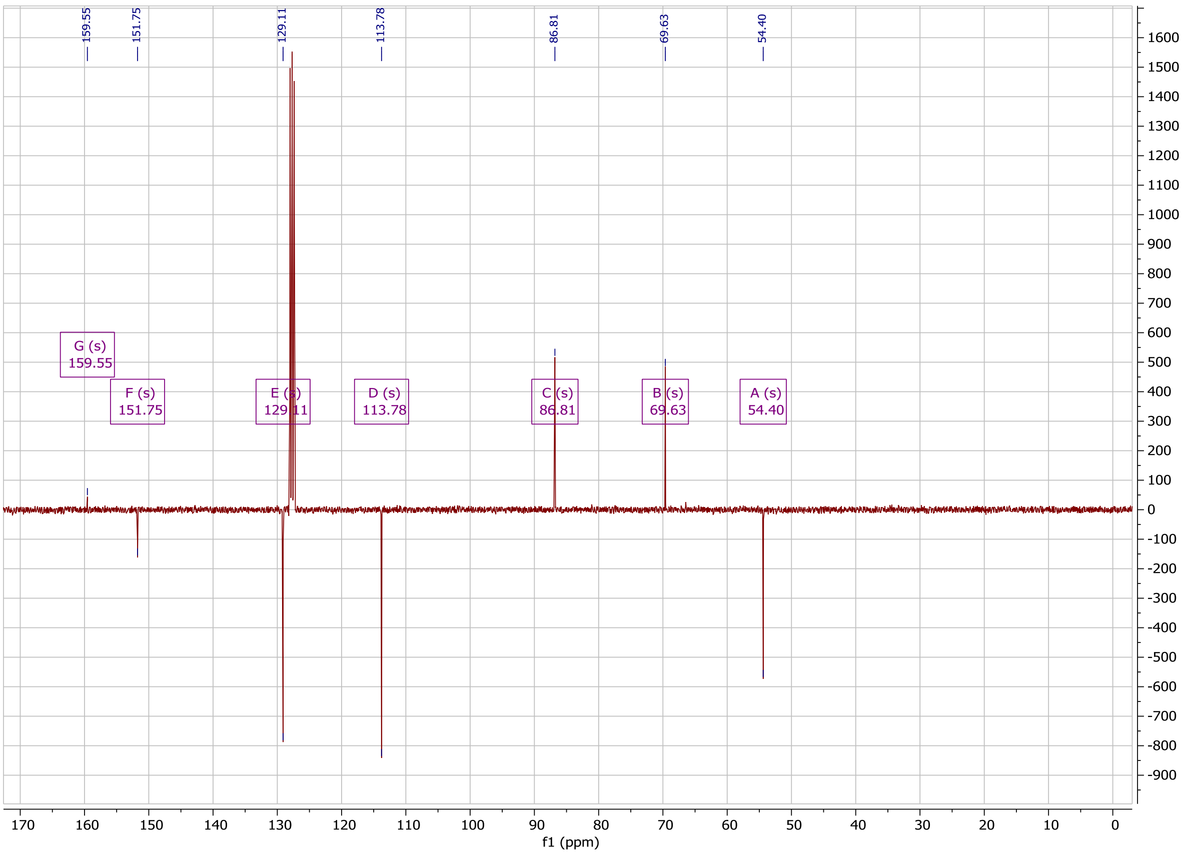

A13

^{1}H NMR (300 MHz, C_6D_6)

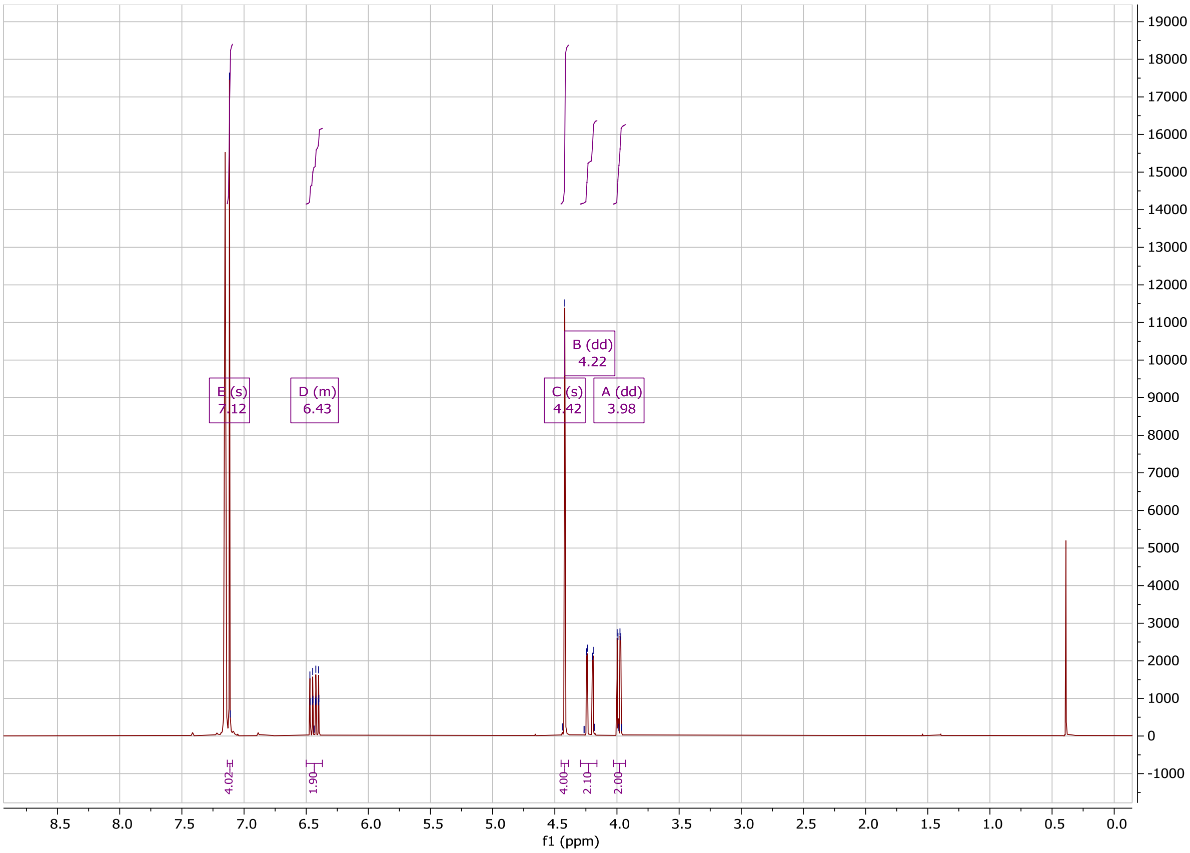

$^{13}C\{^{1}H\}$ NMR(75 MHz, C_6D_6)

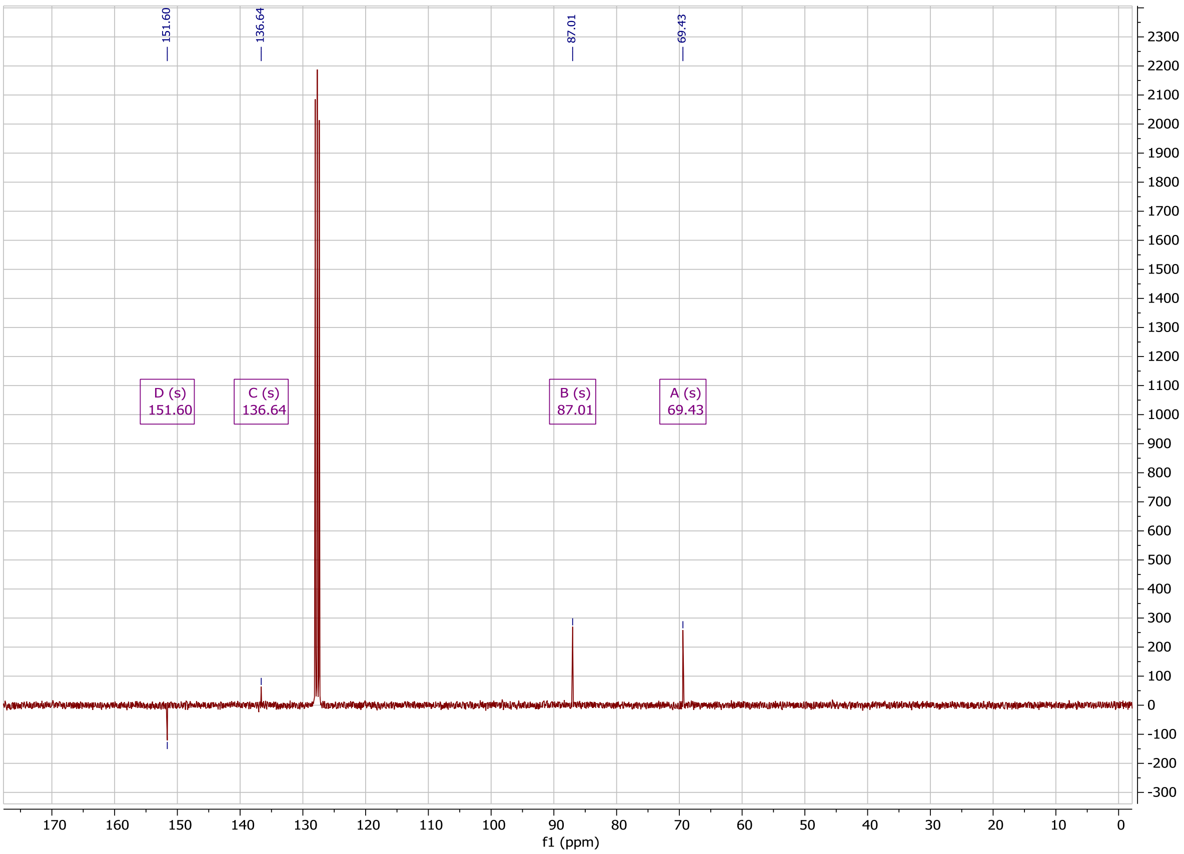

ATR-Ir

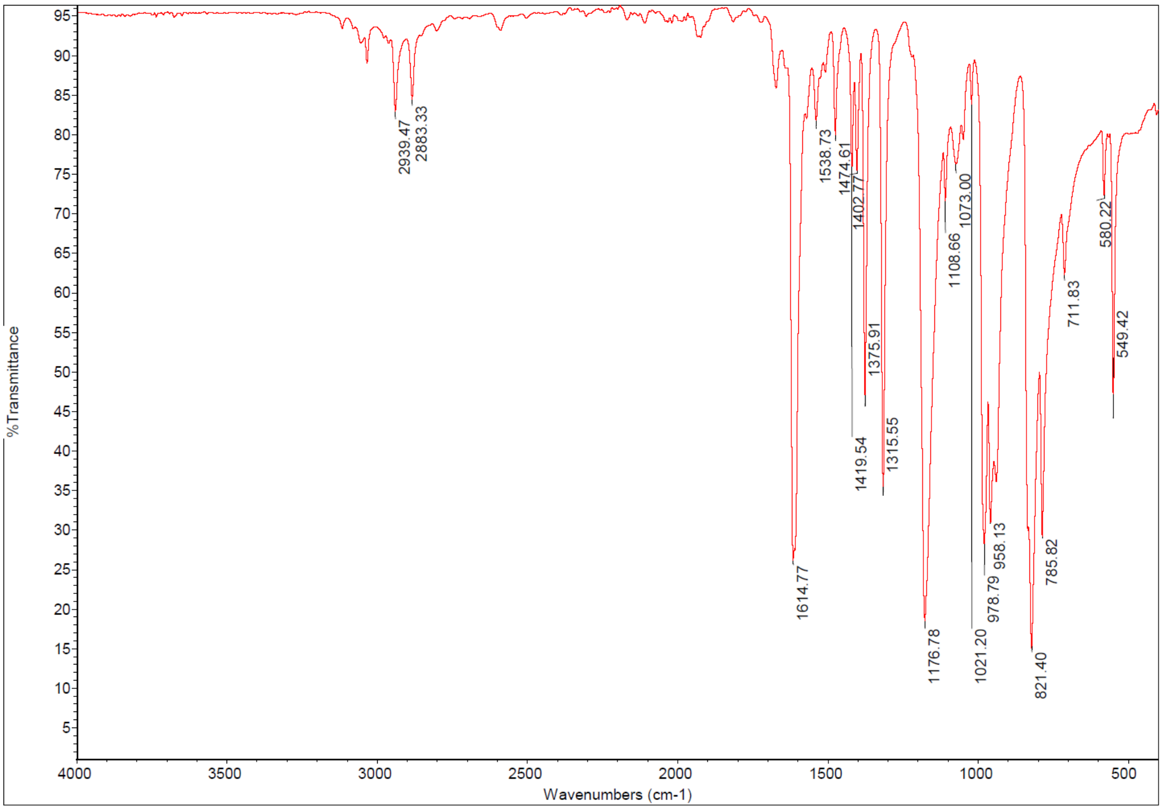

A14

^{1}H NMR (300 MHz, C_6D_6)

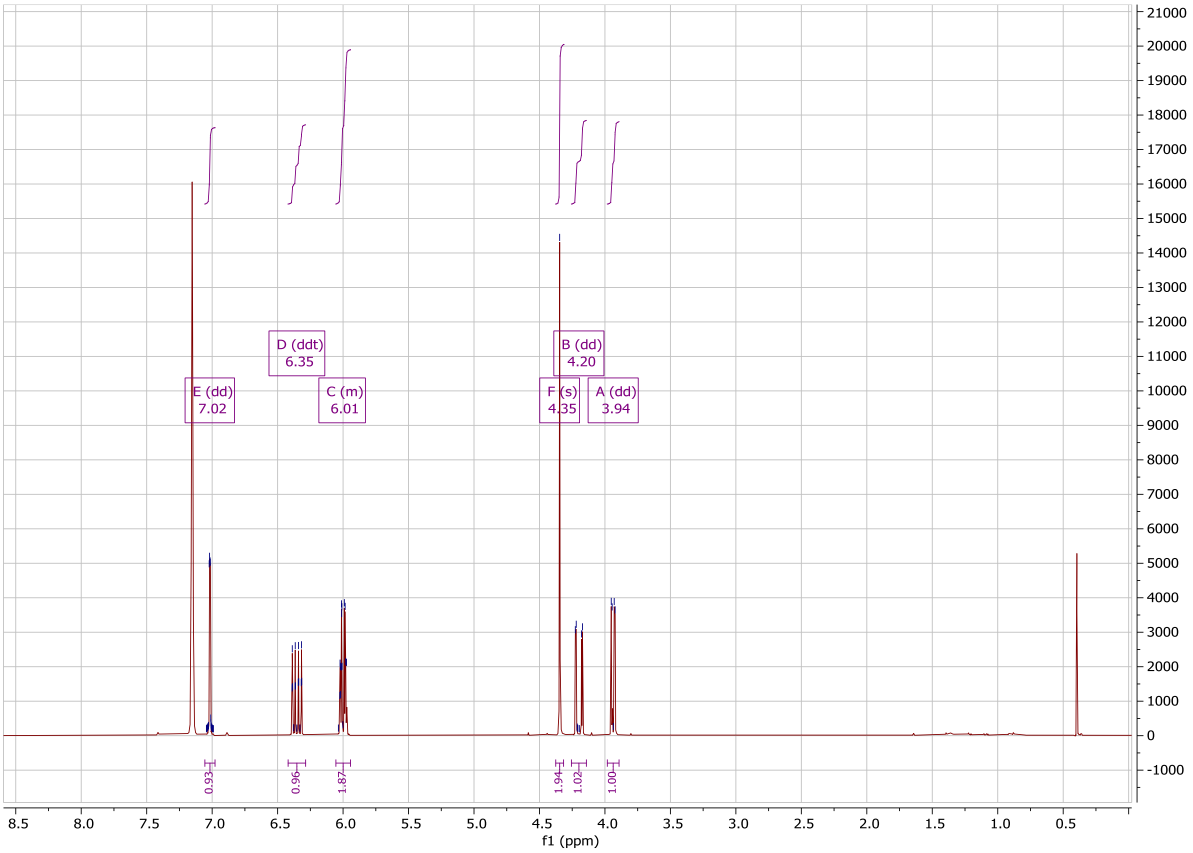

^{13}C{^{1}H} NMR(75 MHz, C_6D_6)

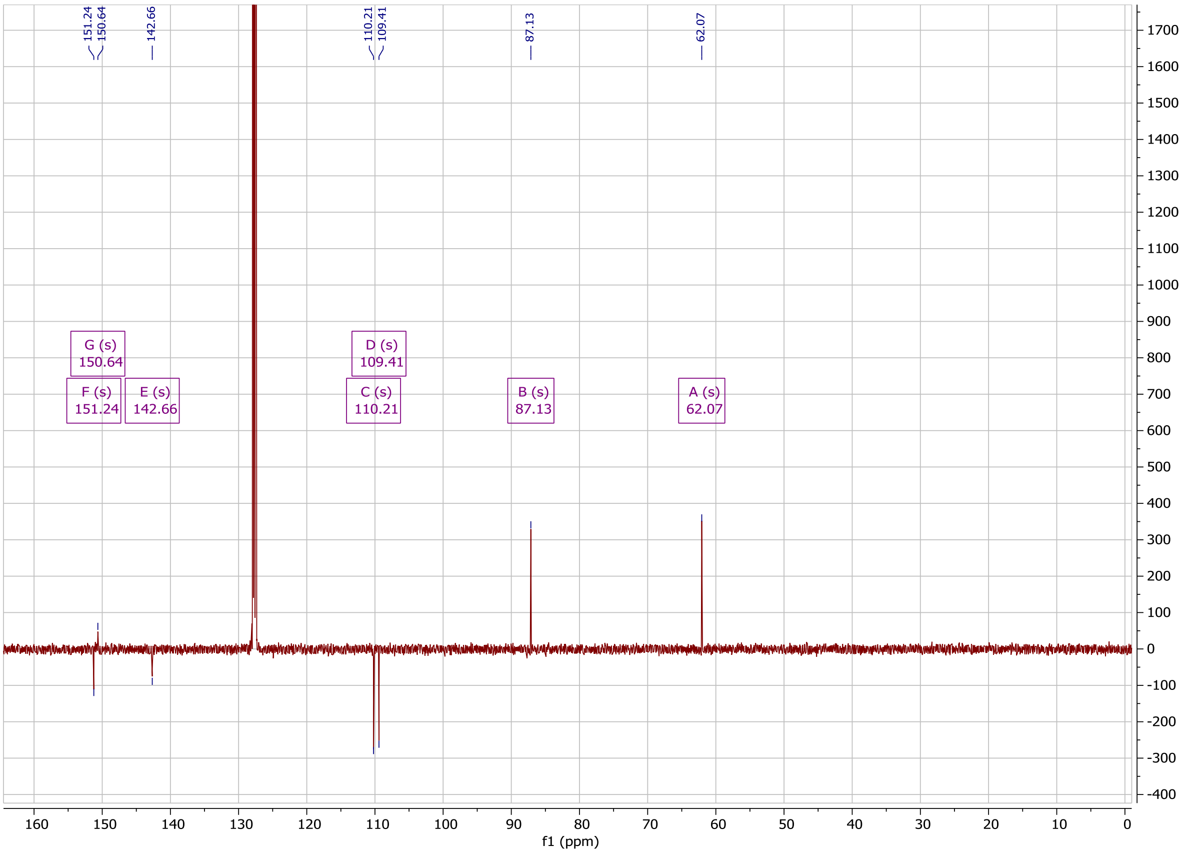

A15

^{1}H NMR (300 MHz, C_6D_6)

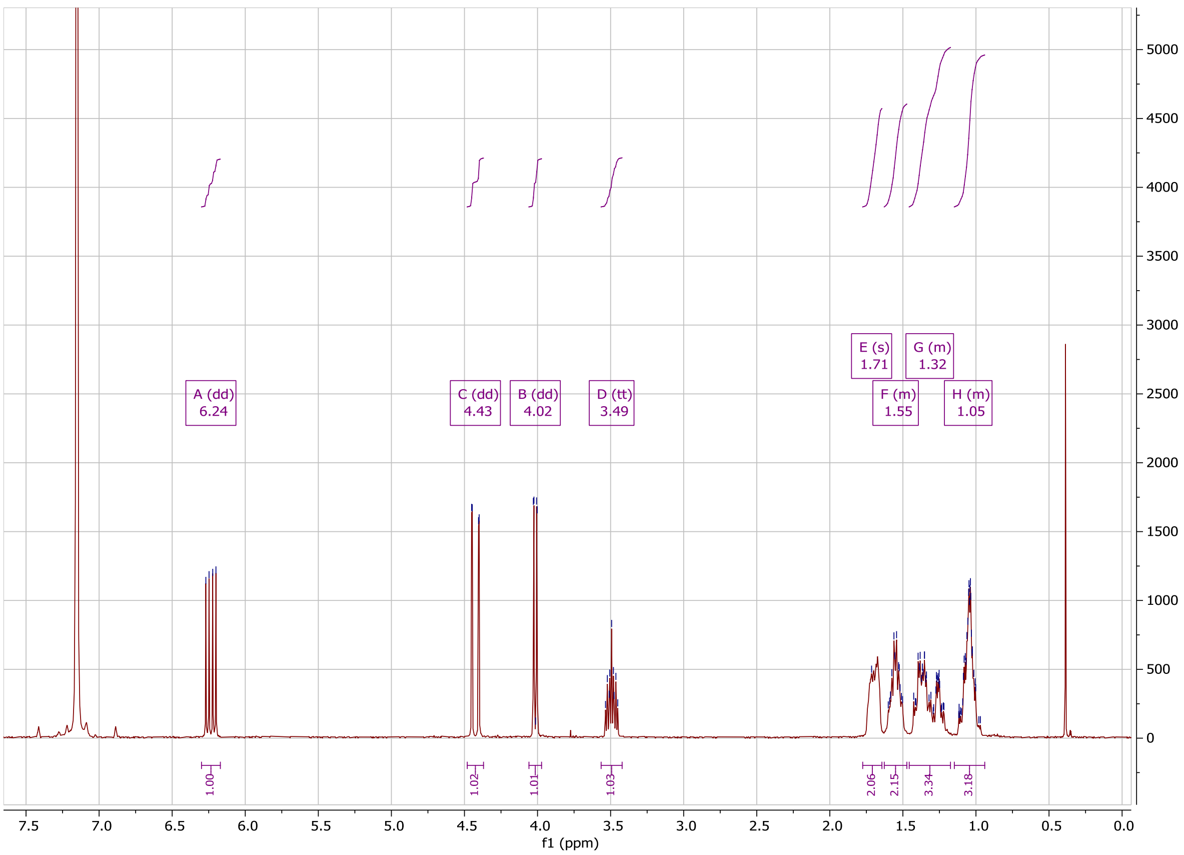

$^{13}C\{^1H\}$ NMR(75 MHz, C_6D_6)

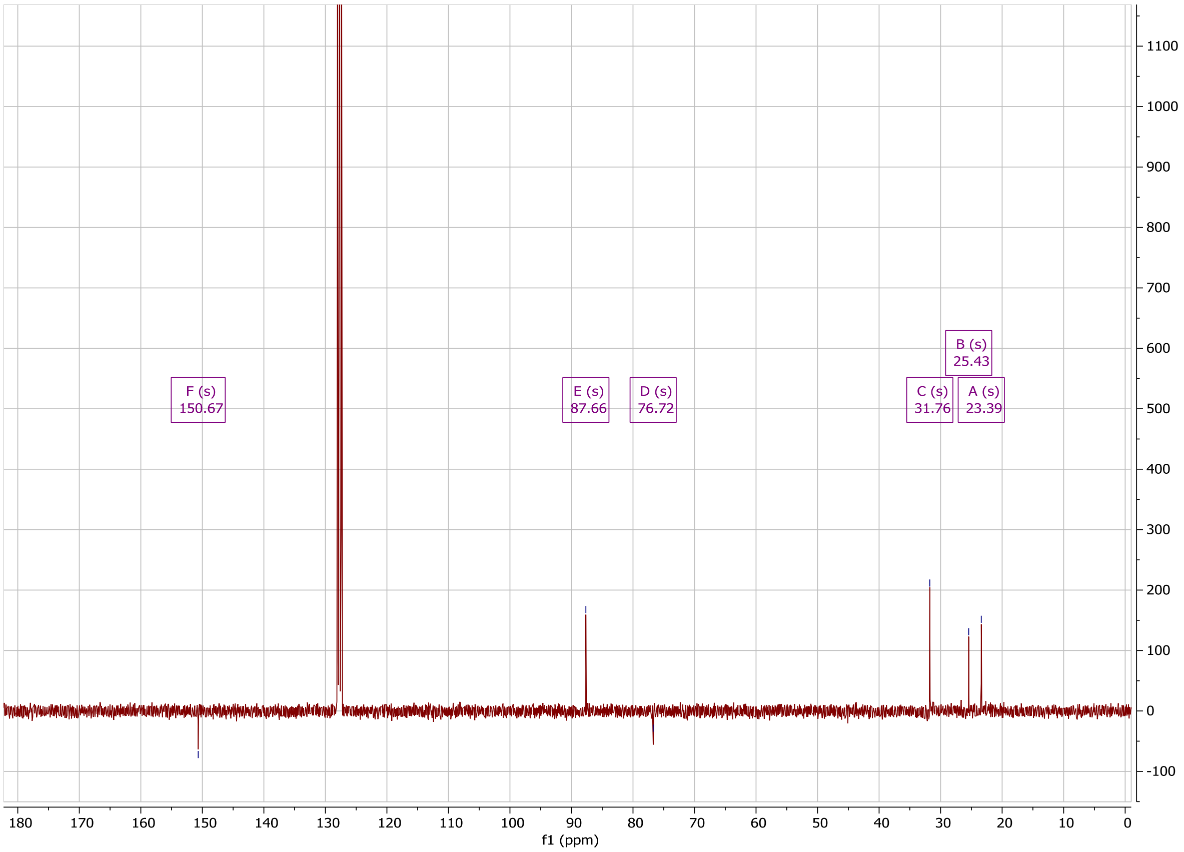

A16

^{1}H NMR (300 MHz, C_6D_6)

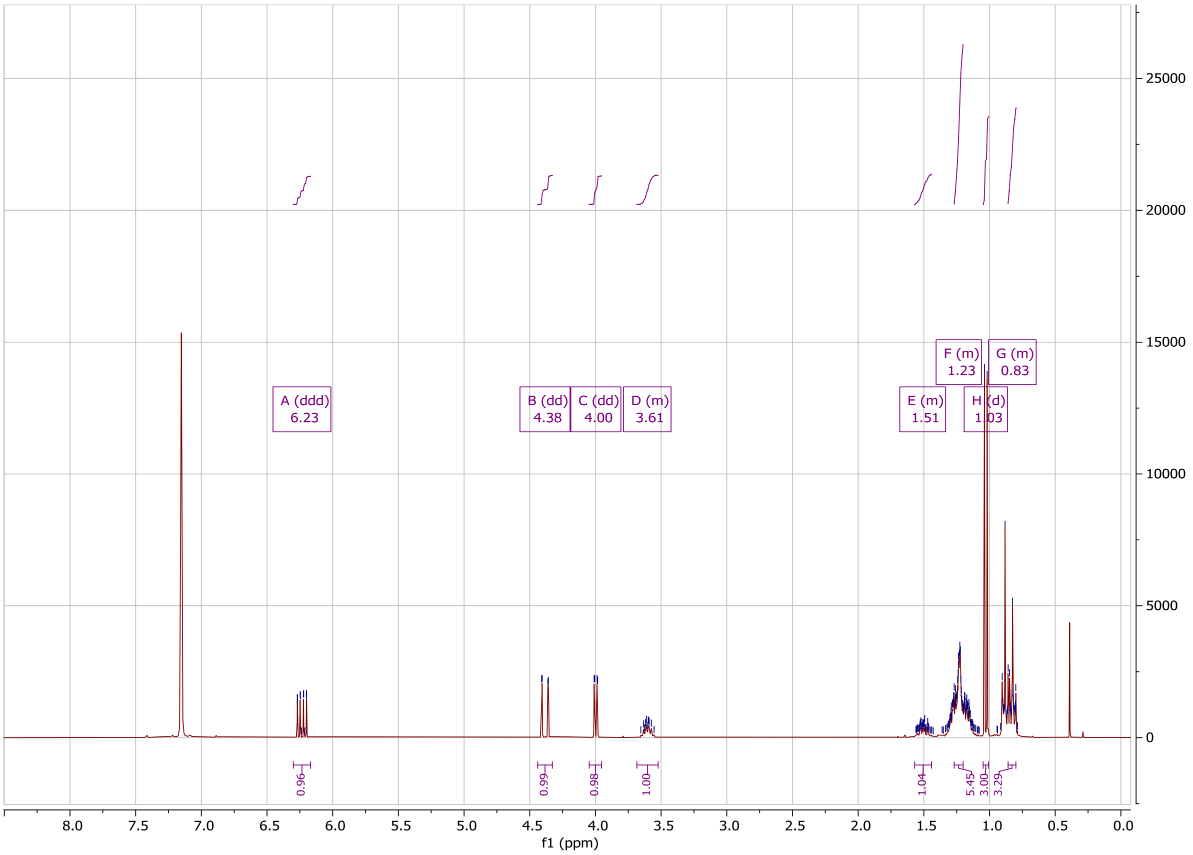

^{13}C{^{1}H} NMR(75 MHz, C_6D_6)

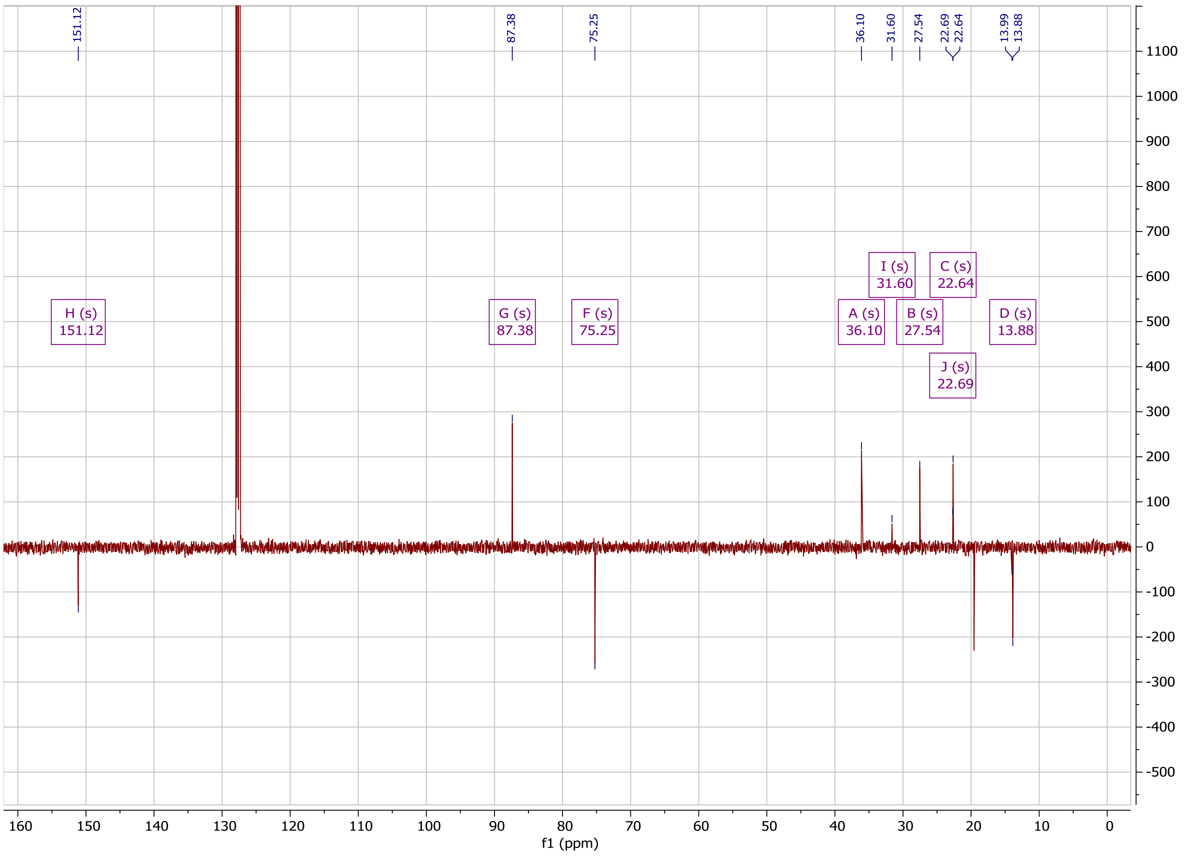

A18

^1H NMR (300 MHz, C_6D_6)

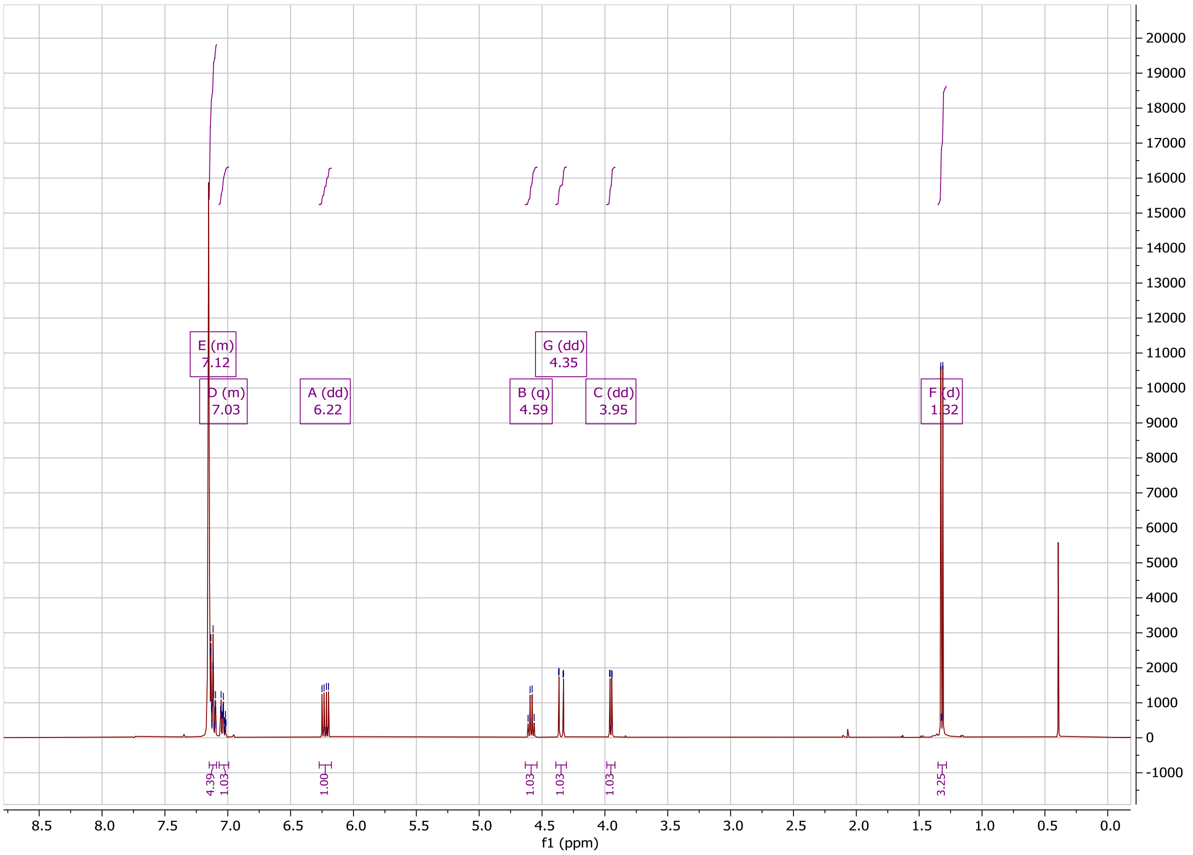

$^{13}C\{^1H\}$ NMR(75 MHz, C_6D_6)

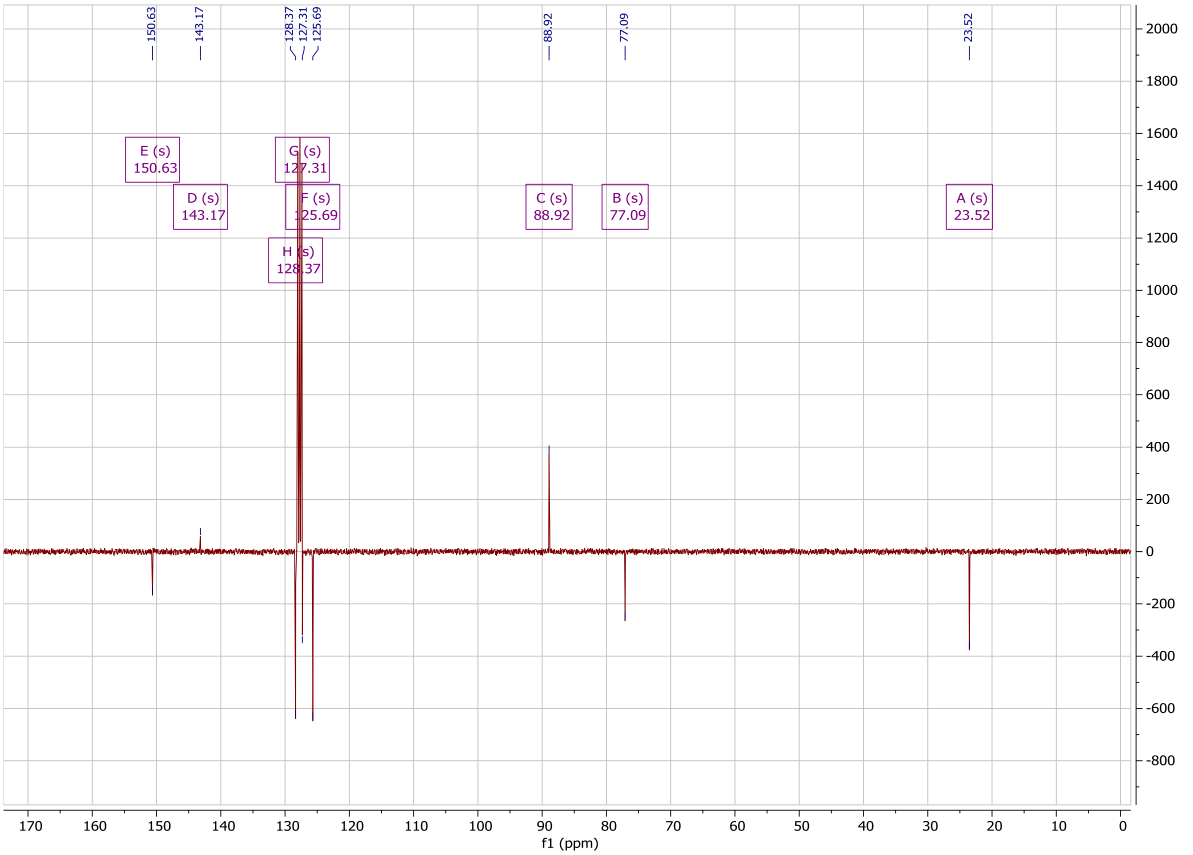

A21

^1H NMR (300 MHz, C_6D_6)

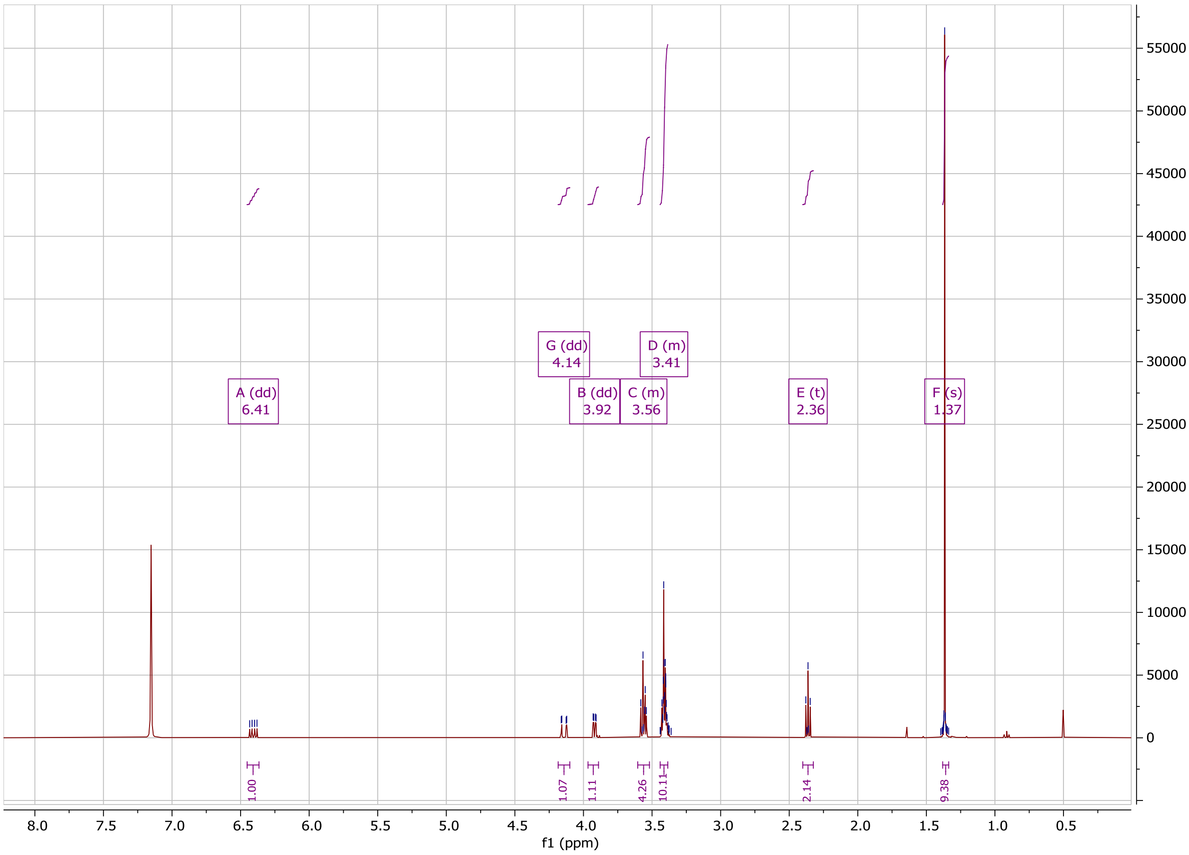

$^{13}C\{^1H\}$ NMR(75 MHz, C_6D_6)

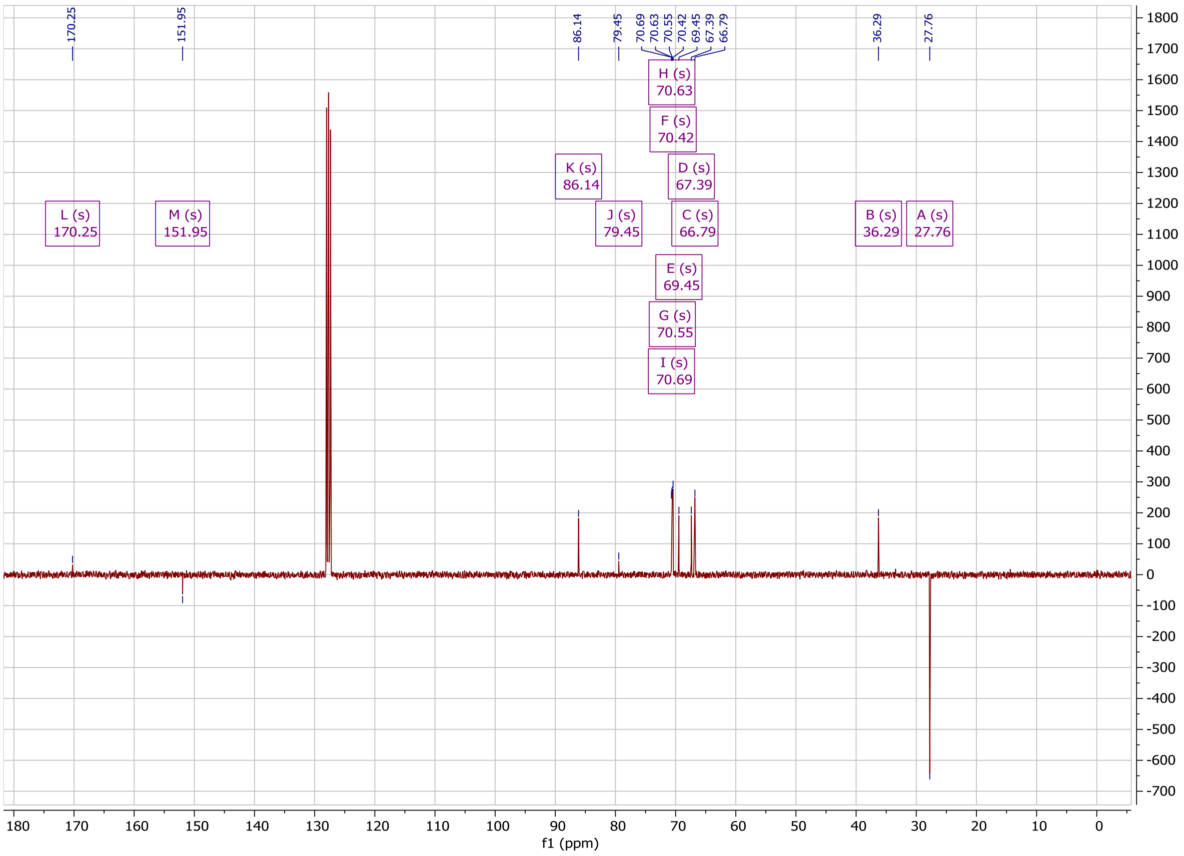

ATR-Ir

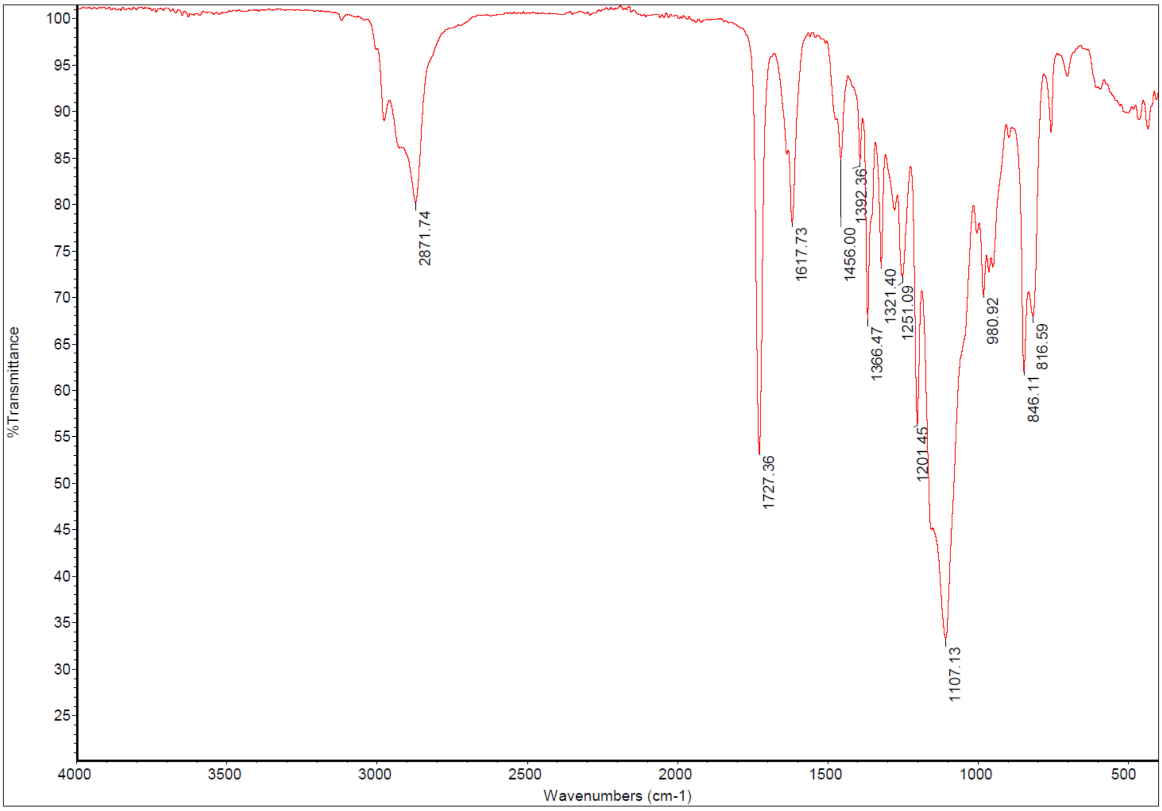

%Transmittance
Wavenumbers (cm-1)
2871.74
1727.36
1617.73
1456.00
1392.36
1366.47
1321.40
1251.09
1201.45
1107.13
980.92
846.11
816.59

3. Additional screening data

3.1. Catalyst screening

Table s1. Results of screening with metals other than Ru (% conversion to BzOVi and remaining BzOH % are shown).

**Metal salt**	**BzOVi/%**
$MeReO_3$	1.38
Re_2O_7	0.53
$Zn(OAc)_2$	0.27
$Zn(OAc)_2*2H_2O$	0.15
Dichlorobis(2,2,6,6-tetramethyl-3,5-heptanedionato)titanium(IV)	0.12
$[Ir(COD)_2]BF_4$	0.00
$Mn(OAc)_2$	0.00
$Fe(OAc)_2$	0.00
$Mn(OAc)_3*2H_2O$	0.00
$Ni(OAc)_2*4H_2O$	0.00
$TiCpCl_3$	0.00
$Zr(OC_2H_5)_4$	0.00
Cyclopentadienyl(p-cymene)ruthenium(II) hexafluorophosphate	0.00
$Zn(TFA)_2$	0.00

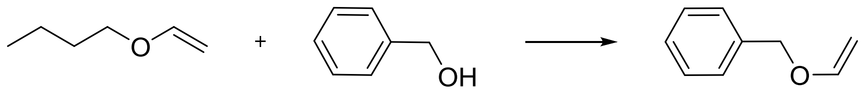

3.2. Phenantroline as a ligand

Table s2. Effect of the addition of 1,10-phenantroline as a ligand (% conversion to BzOVi and remaining BzOH % are shown).

	No ligand	1,10-Phenanthroline
**Metal salt**	**BzOVi/%**	**BzOVi/%**
Bis(2-methylallyl)(1,5-cyclooctadiene)ruthenium(II)	66.30	7.62
$Ru_2Cl_4Cymene_2$	36.55	4.35
$Mn(OAc)_2$	0.00	0.00
$Fe(OAc)_2$	0.00	0.00
$Mn(OAc)_3*2H_2O$	0.00	0.00
$Ni(OAc)_2*4H_2O$	0.00	0.00

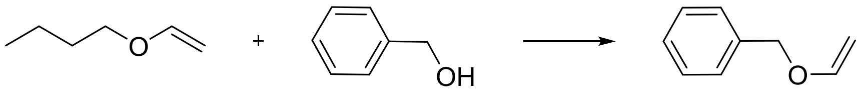

3.3. Ligand screening with **1**

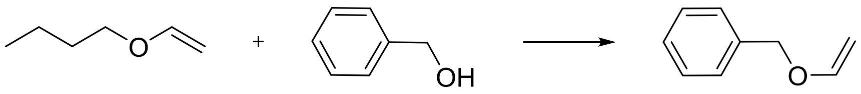

Table s3. Effects of different nitrogen ligands on the catalytic vinylation with **1** (% conversion to BzOVi and remaining BzOH % are shown).

Ligand	BzOVi/%
**No ligand**	66.30
**1,10-phenanthroline**	7.62
**pyridine**	20.96
**4-(4-Nitrobenzyl)pyridine**	6.16
**DMAP**	11.24
**2,4,6-Trimethylpyridine**	60.01
**2,6-lutidine**	67.65
**2-methy-6-phenyl** [illegible]	[illegible]

3.4. Screening with Et_3N

Table s4. Effects of 10 mol% Et_3N on the catalytic vinylation reaction.

	No base	Et_3N 10 mol%
**Catalyst**	**BzOVi**	**BzOVi**
Bis(2-methylallyl)(1,5-cyclooctadiene)ruthenium(II)	66.30	62.92
Hexakis[μ-(acetato-O:O')]-triaqua-μ3-oxotriruthenium(III); (Ruthenium(III) acetate)	49.49	49.18
Dichloro(p-cymene)triphenylphosphineruthenium(II) dichloromethane adduct	42.97	49.93
$Ru_2Cl_4Cymene_2$	36.55	61.99
Dichloro(mesitylene)ruthenium(II) dimer	32.84	54.67
Dichloro(1,5-cyclooctadiene)ruthenium(II) polymer	29.05	10.26
Dichloro(benzene)ruthenium(II) dimer	26.16	33.73
$Ru_2Cl_4Cymene_2$ + DiPh-Phen	5.79	6.55
$Ru_2Cl_4Cymene_2$ + 1,10 Phenantroline	4.35	6.94
Bis(2,2,6,6-tetramethyl-3,5-heptanedionato)(1,5-cyclooctadiene)ruthenium(II	0.90	0.86
Tris(acetonitrile)pentamethylcyclopentadienylruthenium(II) hexafluorophosphate	0.61	1.05
$Zn(OAc)_2$	0.27	0.23
$Zn(OAc)_2*2H_2O$	0.15	0.00
$[Ir(COD)_2]BF_4$	0.00	12.21
Cyclopentadienyl(p-cymene)ruthenium(II) hexafluorophosphate	0.00	0.00

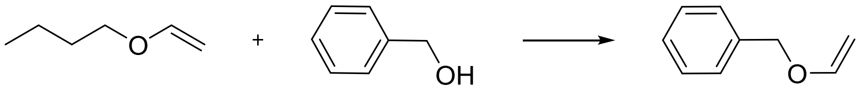

3.5. Purified and unpurified substrate comparison

Table s5. Effects of substrate purification on the catalytic vinylation with **1.**

Compound	Conversion/%	Purified Conversion/%
Anisyl alcohol	93	82
Benzyl alcohol	76	84
N-(3-Hydroxypropyl)carbamic acid tert-butyl ester	54	67
1-octanol	68	>98
4-Chlorobenzyl alcohol	64	92
2-hexanol	61	58
Furfuryl alcohol	60	92
1-phenylethanol	54	41
3-hexanol	54	42
5-Hydroxy-2-pentanone, mono+dimer	48	48
Diphenylmethanol	44	44
4-(Trifluoromethyl)benzyl alcohol	20	>98
1,4-Benzenedimethanol	10	76
3,5-dichlorobenzyl alcohol	4	>98
4-Hydroxybenzyl alcohol	4	0
4-Hydroxybenzyl alcohol	4	0
Boc-Thr-OMe	0	0
6-Mercapto-1-hexanol	0	0
2-(4-Hydroxyphenyl)ethanol	0	30
trans-p-Menth-6-ene-2,8-diol	0	4
4-methoxyphenol	0	4
2-methyl-2-hexanol	0	0

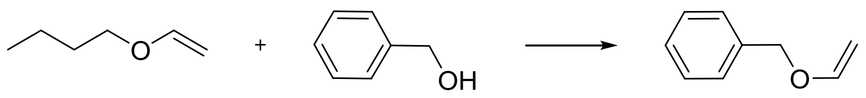

3.6. Comprehensive table of conversions with **1** and unpurified substrates

Table s6. Conversions using **1** with unpurified substrates.

ompound	Conversion/%	Compound	Conversion/
(Boc-amino)-1-hexanol	>98	3-Methyl-1,3-butanediol	14
dodecanol	>98	1,4-Benzenedimethanol	10
hexanol	>98	trans-1,2-Cyclohexanediol	8
rt-Butyl 12-hydroxy-4,7,10-trioxadodecanoate	>98	N-Boc-Valinol	7
fluorobenzyl alcohol	96	3,5-dichlorobenzyl alcohol	4
isyl alcohol	93	4-Hydroxybenzyl alcohol	4
Butanol	88	Methyl (S)-(-)-lactate	2
clohexanol	80	phenol	0
nzyl alcohol	76	6-Mercapto-1-hexanol	0
(3-Hydroxypropyl)carbamic acid tert-butyl ester	54	2-Hydroxybenzonitrile	0
octanol	68	glycoamide	0
rt-Butyl 3-hydroxypropionate	68	2-(4-Hydroxyphenyl)ethanol	0
Chlorobenzyl alcohol	64	Boc-Thr-OMe	0
hexanol	61	3-Hydroxypropionitrile	0
6-dichlorobenzyl alcohol	60	6-hepten-1ol	0
rfuryl alcohol	60	2-(Benzylthio) ethanol	0
phenylethanol	54	3-Methyl-4-nitrobenzyl alcohol	0
hexanol	54	6-amino-1-hexanol	0
Menthol	49	tert-Butyl alcohol	0
Hydroxy-2-pentanone, mono+dimer	48	1,2-Decanediol	0
phenylmethanol	44	1,2-Dodecanediol	0
ethyl 4-(hydroxymethyl)benzoate	44	4-Hydroxy-4-methyl-2-pentanone	0
4-Cyclohexanedimethanol, cis + trans	44	cis-1,2-cyclohexanediol	0
peronyl alcohol	36	1-phenyl-1,2-ethanediol	0
penten-1-ol	32	2-methyl-2-hexanol	0
ycidol	24	2-methyl-2-butanol	0
(Trifluoromethyl)benzyl alcohol	20	4-methoxyphenol	0
Boc-ethanolamine	17	trans-p-Menth-6-ene-2,8-diol	0
ethyl (R)-(-)-3-hydroxybutyrate	16	Boc-Ser-OMe	0
4-butane diol	15		

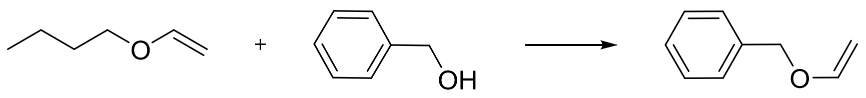

3.7. Other screening data

3.7.1. Primary amine addition

21

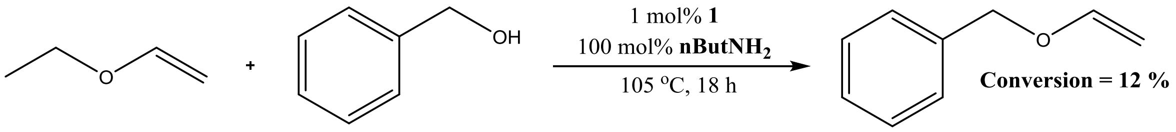

4. Conversion determination - example

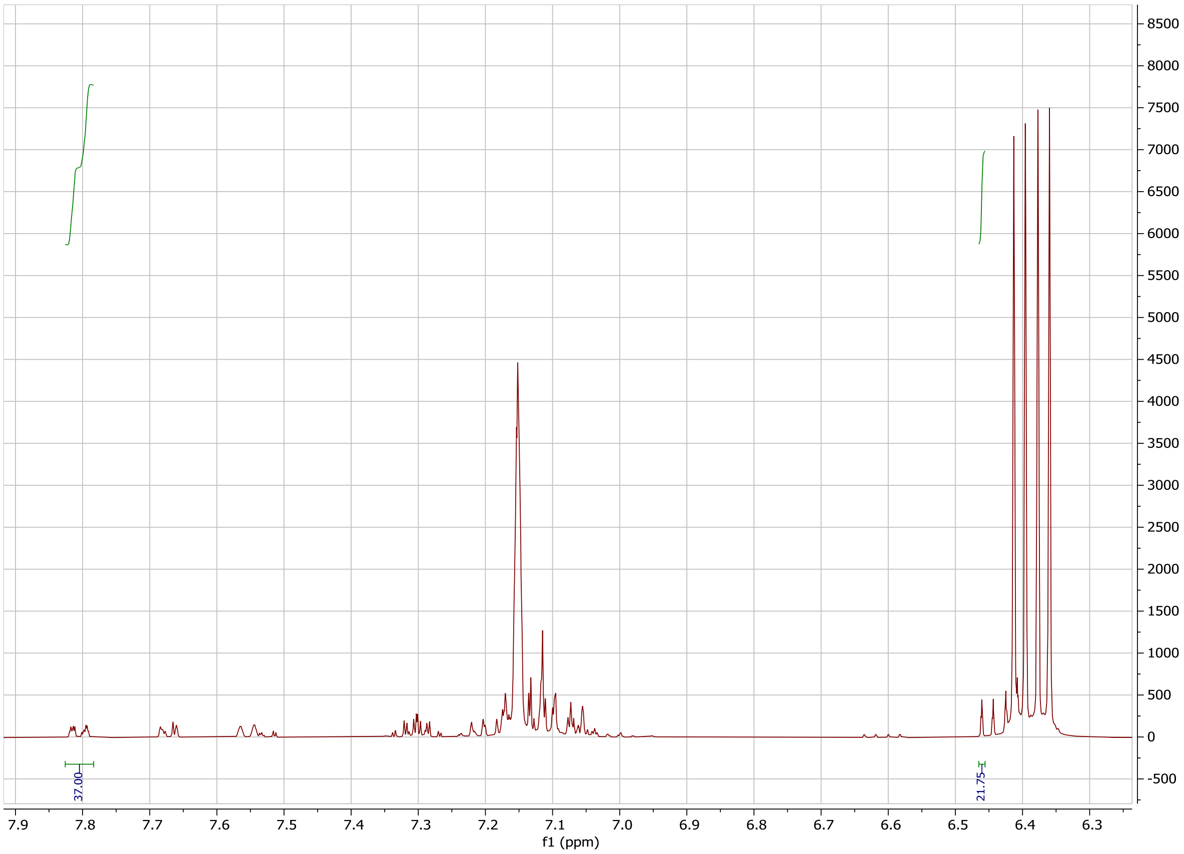

Figure s1. An example of the method used to determine conversion. The methyl naphthalene signal is set to 37 (equal to mmol*10 of methyl naphthalene in the reaction mixture) and compared to one of the peaks in the dd signal of the product. Since there is 1 mmol of substrate in the reaction mixture conversion is equal to $I_{peak}/25*100$. The example shown is for benzyl alcohol vinylation.

5. Substrate scope – additional examples

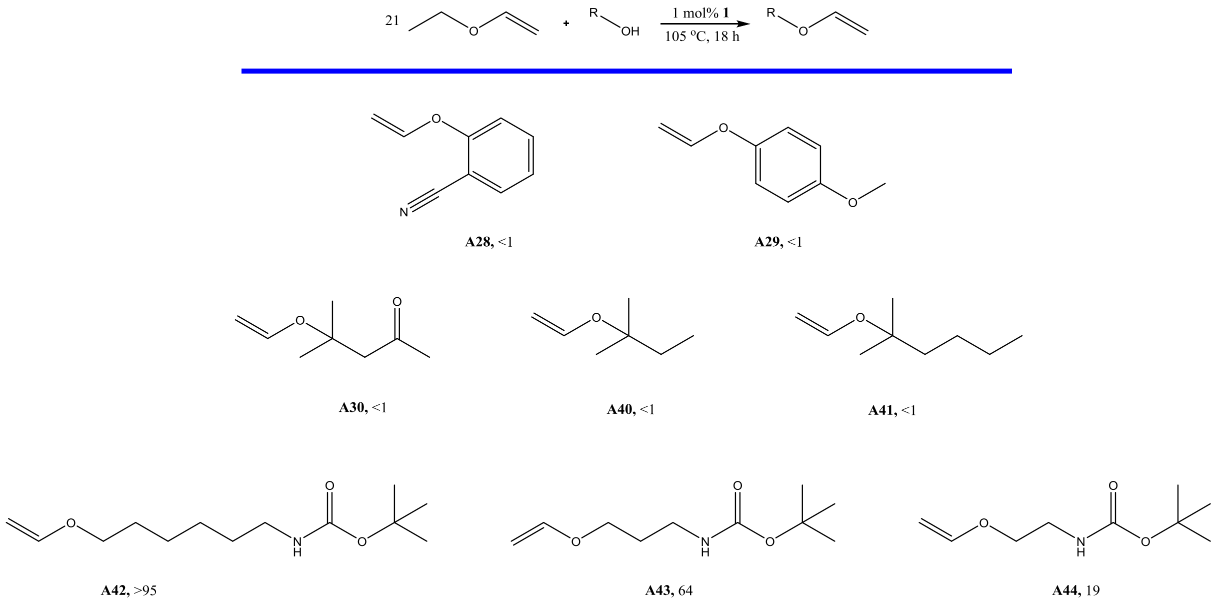

Scheme s1. Transfer vinylation of different alcohols by ethyl vinyl ether in the presence of catalyst **1**.

6. Recovery and Reutilization of Organic Reaction Materials.

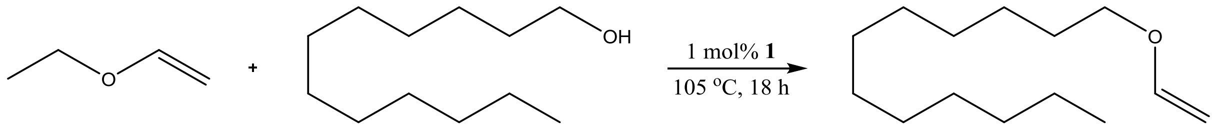

m(Dodecanole)= 12.577 g (67.495 mmol)

m(**1**) = 224 mg

m(EtOVi) = 105.458 g (1462.541 mmol)

Results:

m(product, theoretical yield) = 14.335 g
m(product, technical purity)= 13.1 g (91%)
m(product, pure)= 11.257 g (79%)

m(EtOVi, theoretical recovery yield) = 100.591 g
m(EtOVi, isolated) = 81.42 g (81 %)

m(EtOH, theoretical amount) = 3.11 g
m(EtOH, detected amount) = 2.788 g (90 %)
m(EtOH, isolated water solution) = 2.46 g (79 %)

Procedure:

Reaction was set up in a oven dried pressure flask under argon, solids were added in a glovebox and liquids by syringe under argon flow. After 18h the reaction vessel was cooled down and extraction was performed with a saturated brine solution (5 x 35 g of saturated brine). The layers were separated, and the organic layer was submitted to distillation at 62 °C. The distilled EtOVi submitted to extraction with brine (2 x 22 g of saturated brine) and dried with sodium sulphate, affording pure EtOVi.

The remaining organic layer was used for kugelrorh distillation at 80°C and Schlenk line vacuum affording the product in technical purity.

To further purify the product a filtration column with 90 g of silica was set up. The product was introduced to the dry column and washed down with hexane (150 ml), until the parent alcohol started eluting. Hexane was evaporated affording the pure product.

All the brine solutions from the experiment were combined and distilled to recover ethanol. The concentration of ethanol was determined from NMR in D_2O by using sodium acetate as a mass standard. Two fractions were distilled off: 1) 3.617 g of 27 % ethanol in water (0.99 g ethanol), 2) 4.6 g of 33 % ethanol in water (1.47 g ethanol). The ethanol in the remaining brine solution from distillation was quantified by the same method (329 mg ethanol in 196 g brine solution).

6.1. Recovered ethyl vinyl ether ^{1}H NMR spectra (300 MHz, C_6D_6)

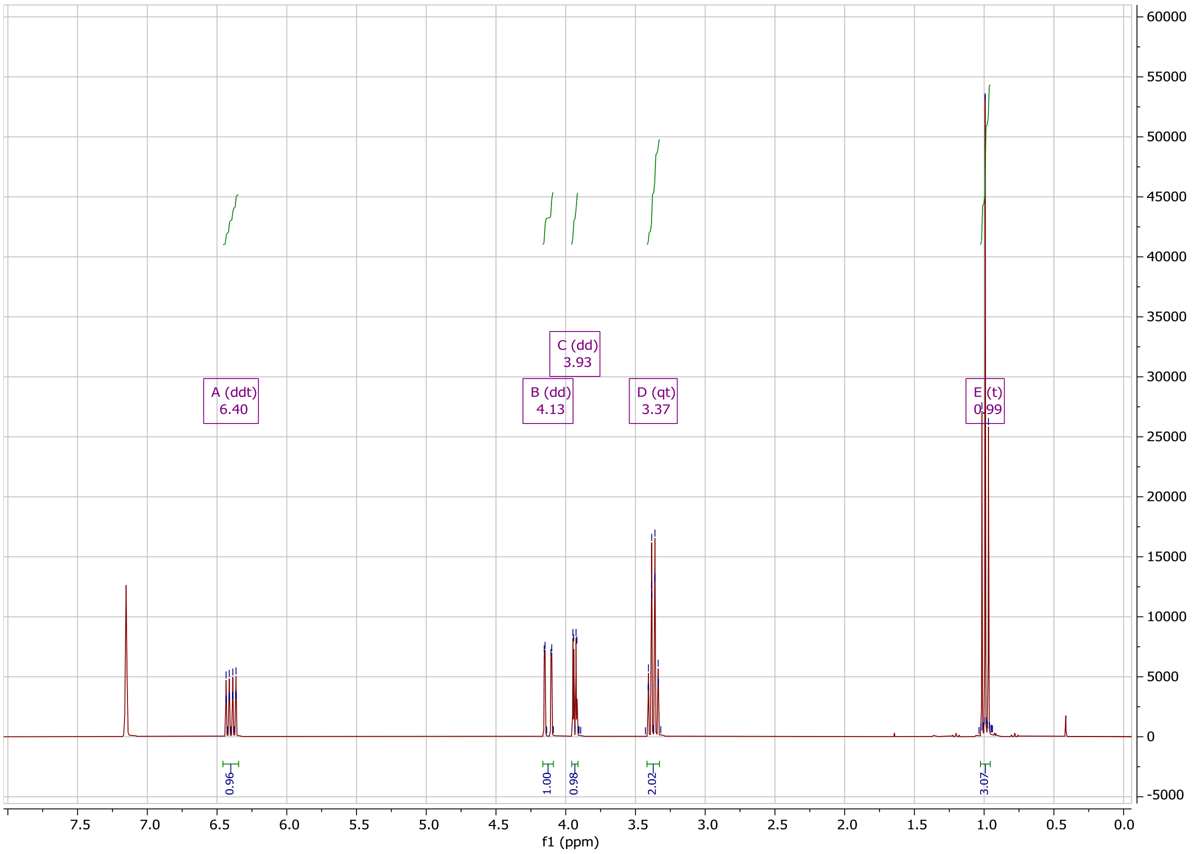

6.2. Product in technical purity ^{1}H NMR spectra(300 MHz, C_6D_6)

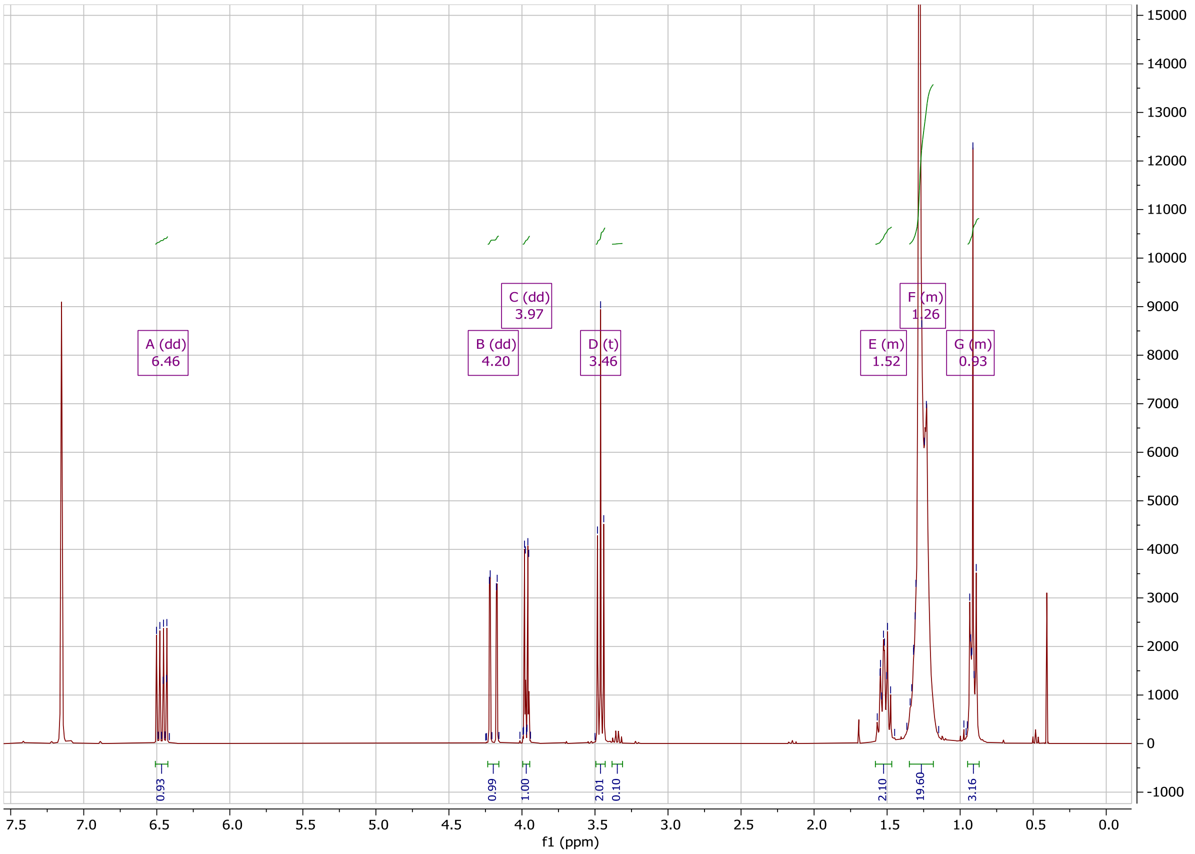

6.3. Pure product ^{1}H NMR spectra (300 MHz, C_6D_6)

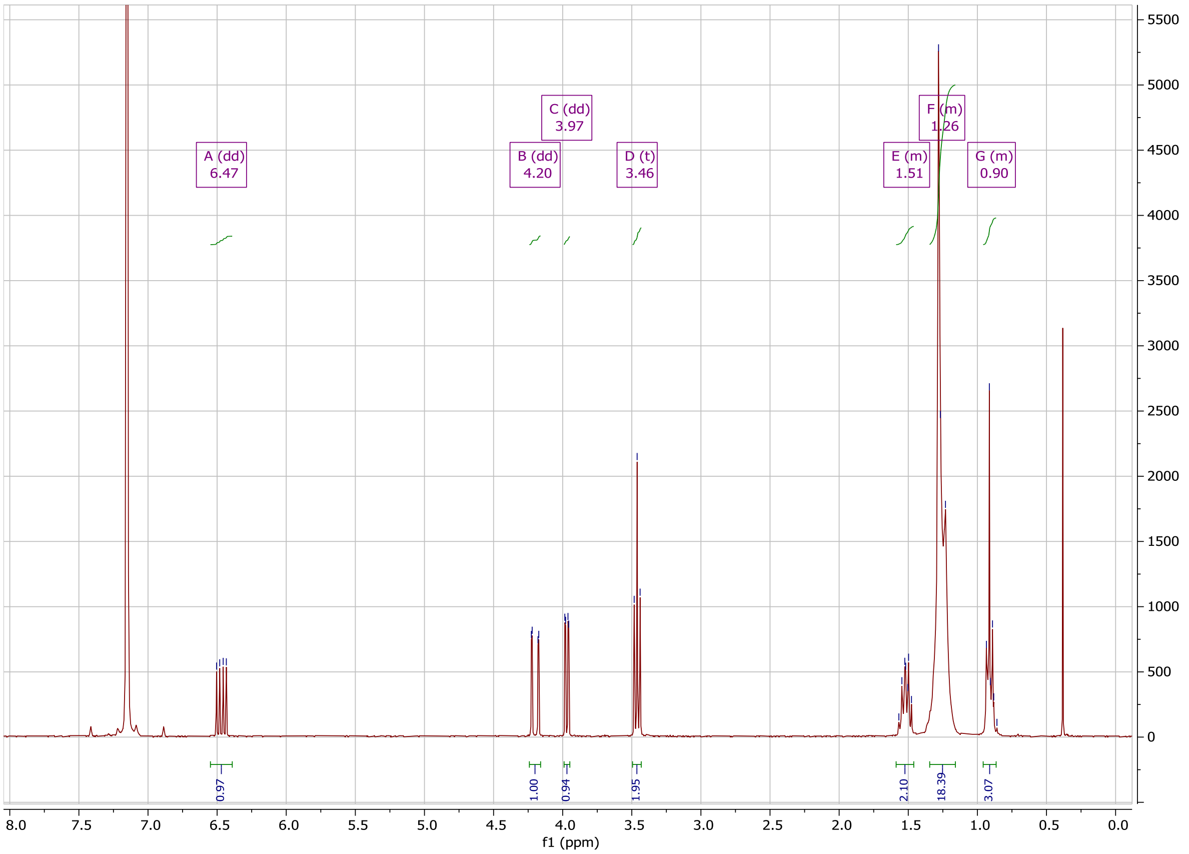

6.4. Brine ^{1}H NMR spectra (300 MHz, D_2O)

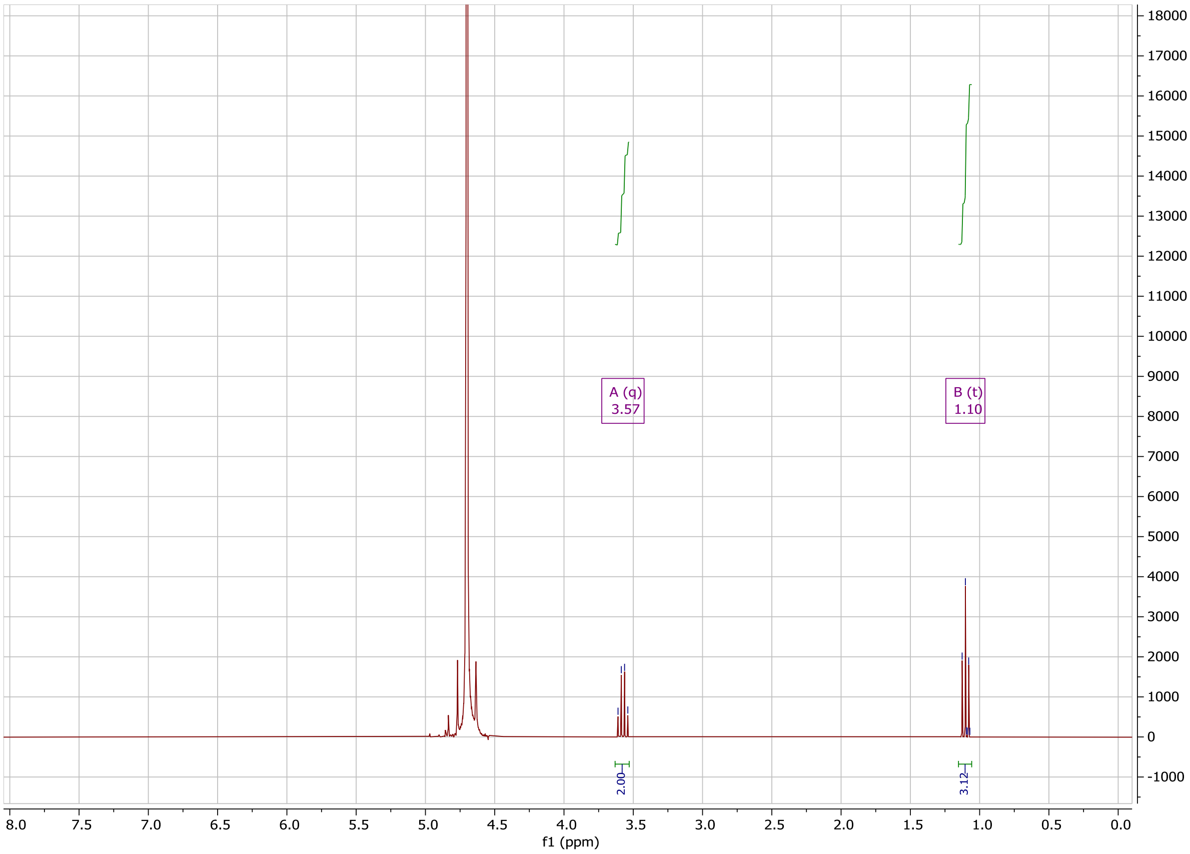

6.5. Recovered ethanol ^{1}H NMR spectra (300 MHz, D_2O)

6.5.1. Fraction 1

Contains 3.8 mg of sodium acetate and 8.8 mg of sample

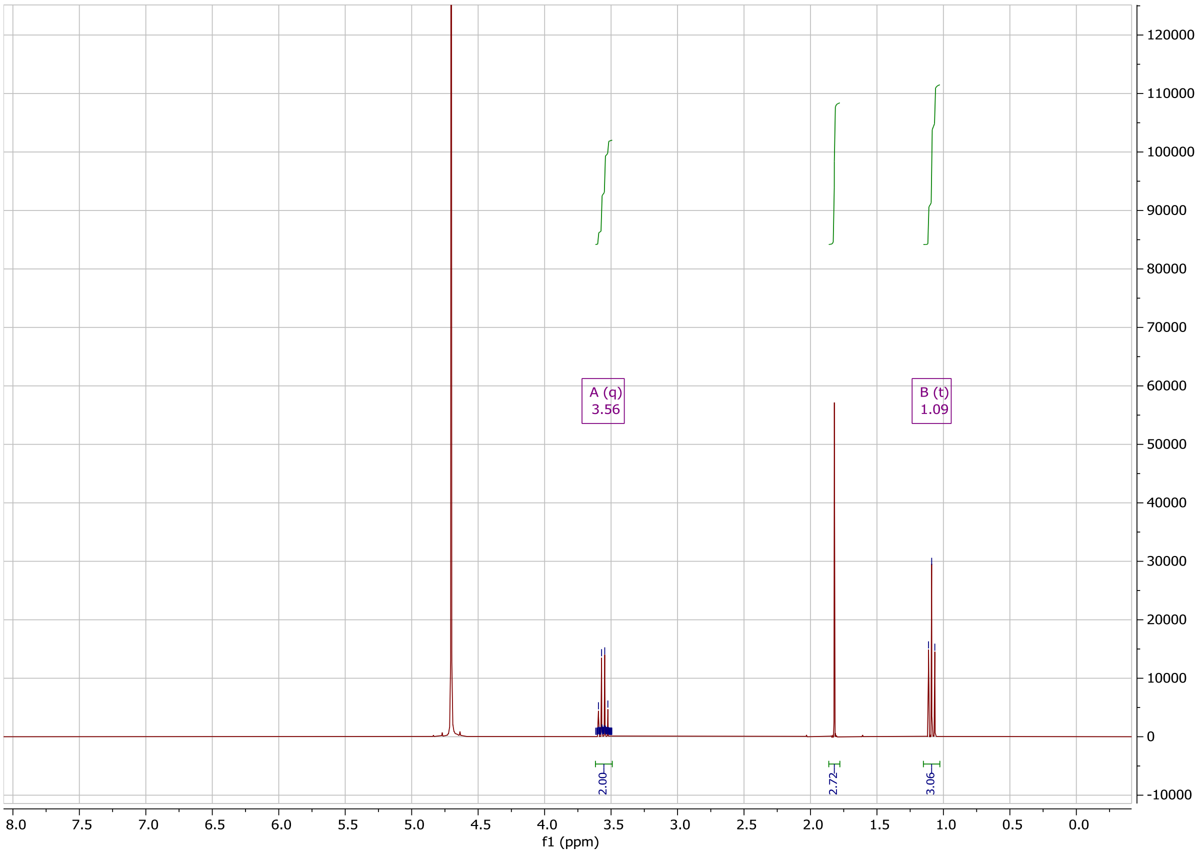

6.5.2. Fraction 2

Contains 5 mg of sodium acetate and 8.8 mg of sample

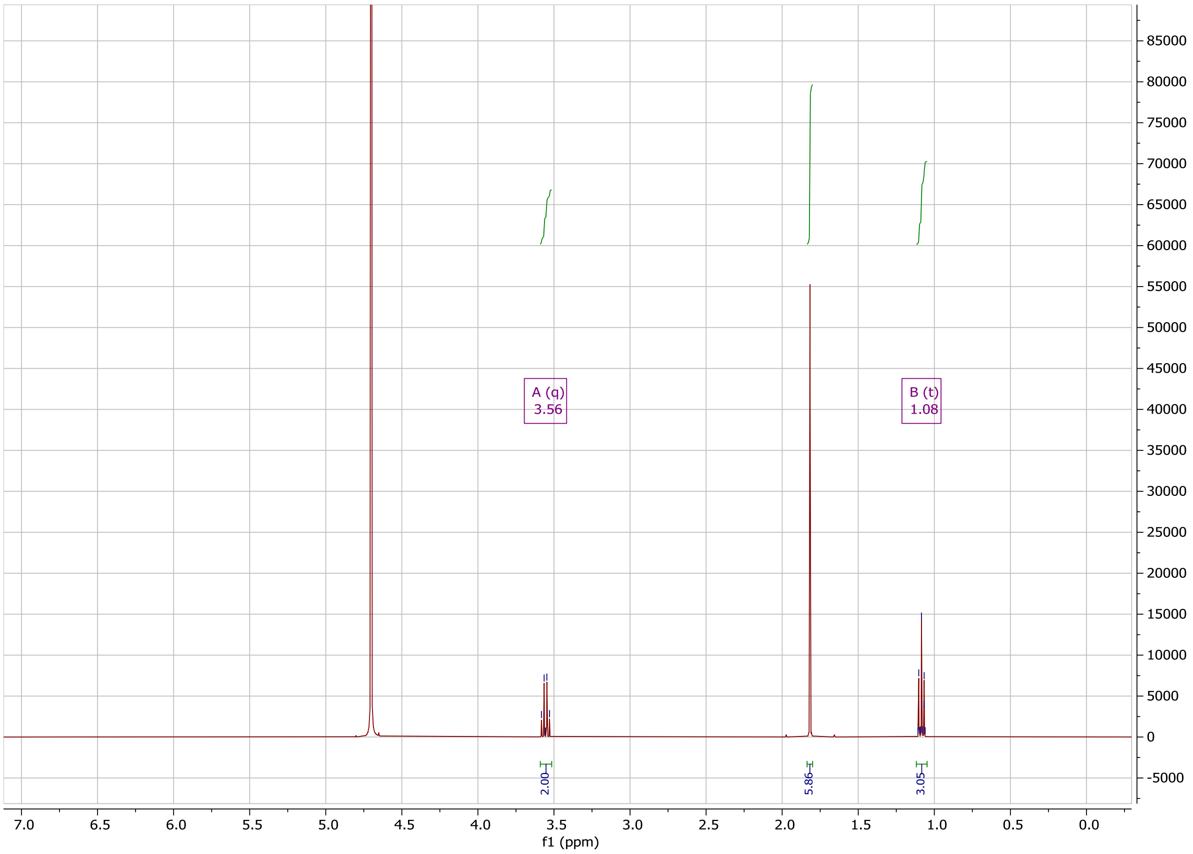

6.5.3. Remaining brine solution

Contains 7.6 mg of sodium acetate and 56 mg of sample

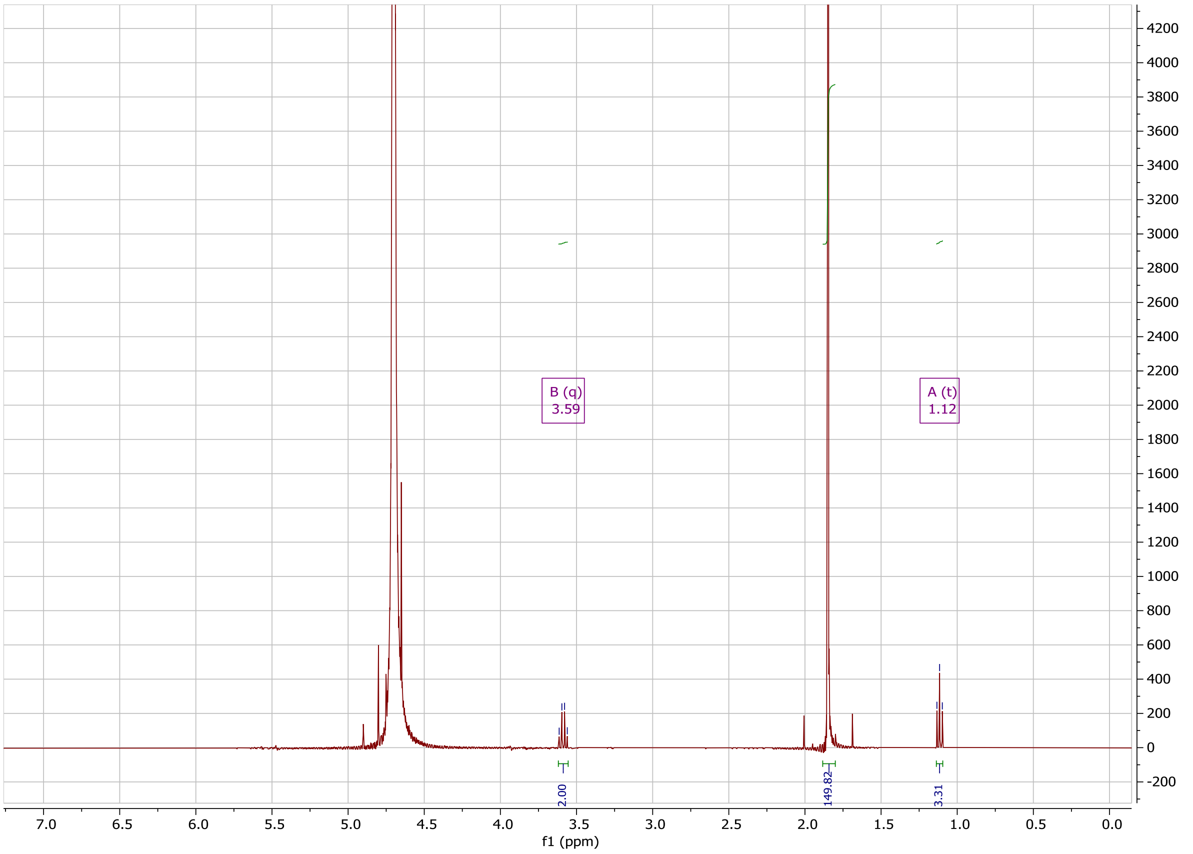

7. Vinyl acetate

7.1. General procedure

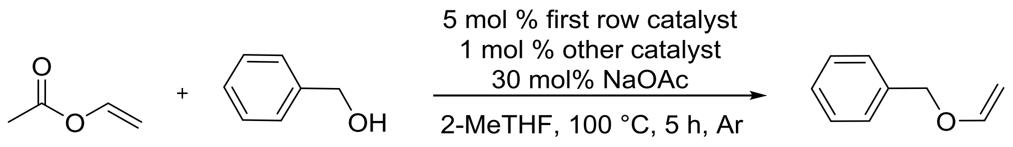

Solids (**catalyst** – 5 mol% for first row transition metals 1 mol% for others, **NaOAc** – base, 30 mol%) were added to a pressure flask in a glovebox. A stock solution was made containing **2-MeTHF** (solvent, distilled), **benzyl alcohol** (substrate, distilled, deggased), **vinyl acetate** (vinyl group source, 3 eq., distilled, deggased) and hexadecane (Gc mass standard, dried over mol sieve) and added with a syringe under argon flow. Sealed tubes were put in an aluminium heating mantle and heated to 100 °C with magnetic stirring for 5 h. After the reaction the tubes were cooled down, their contents filtered through a small silica plug, eluted with EtOAc and the conversions were measured by Gc.

7.2. Catalyst and condition screening

Table s7. Screening of various catalysts for the vinylation reaction with vinyl acetate

Catalyst (additional conditions)	BzOVi	BzOAC
$[Ir(COD)_2]BF_4$	76.5	1.2
$Ni(COD)_2$	0	43
$Mn(OAc)_2$	0	0
$Ru_2Cl_4Cymene_2$	0	0
$Fe(OAc)_2$	0	13.5
Bis(2,2,6,6-tetramethyl-3,5-heptanedionato)(1,5-cyclooctadiene)ruthenium(II	0	0
Hexakis[μ-(acetato-O:O')]-triaqua-μ3-oxotriruthenium(III); (Ruthenium(III) acetate)	0	0
$Mn(OAc)_3$*$2H_2O$	0	0
$Ni(OAc)_2$*$4H_2O$	0	0
Bis(2-methylallyl)(1,5-cyclooctadiene)ruthenium(II)	0	0
$TiCpCl_3$	0.00	67.70
$ZrCl_4$	0.00	55.00
$[Rh(COD)_2]BF_4$	0.28	6.20
$Zr(OC_2H_5)_4$	0	64.36
Dichlorobis(2,2,6,6-tetramethyl-3,5-heptanedionato)titanium(IV)	0	27.79
$ZrCpCl_3$	0	54.13
$Zr(Ot\text{-}But)_4$	0	73.06
Dichloro(mesitylene)ruthenium(II) dimer	0.38	4.09
Dichloro(benzene)ruthenium(II) dimer	0.73	3.36
Dichloro(p-cymene)triphenylphosphineruthenium(II) dichloromethane adduct	0.46	2.42
Dichloro(1,5-cyclooctadiene)ruthenium(II) polymer	0.00	2.18
Cyclopentadienyl(p-cymene)ruthenium(II) hexafluorophosphate	0.00	5.19
Tris(acetonitrile)pentamethylcyclopentadienylruthenium(II) hexafluorophosphate	0.00	1.77
$Au(PPh_3)Cl$ (2mol%)	0.00	3.38
$Au(PPh_3)Cl$ + AgOAc (2mol%)	0.00	27.93

Table s8 Screening of catalysts for the vinylation reaction with vinyl acetate. 1,10 phenantroline (phen), 1,2-Bis(diphenylphosphino)ethane (DPPE), Bis(diphenylphosphino)methane (DPPM)

Ligand		Phenantroline		DPPE		DPPM
**Catalyst (additional conditions)**	**BzOVi**	**BzOAC**	**BzOVi**	**BzOAC**	**BzOVi**	**BzOAC**
$[Ir(COD)_2]BF_4$	2.1	1.5	5.3	1.7		
$Ni(COD)_2$			0	43	0	40
$Co(OAc)_2$	0	84.5	0	52	0	80
$Mn(OAc)_2$	0	76.5	0	28		
$Ru_2Cl_4Cymene_2$	0	23.5			0	0
$Fe(OAc)_2$	0	96	0	29		
Bis(2,2,6,6-tetramethyl-3,5-heptanedionato)(1,5-cyclooctadiene)ruthenium(II			0	2.5		
Hexakis[μ-(acetato-O:O')]-triaqua-μ3-oxotriruthenium(III); (Ruthenium(III) acetate)			0	10		
$Mn(OAc)_3 {*} 2H_2O$			0	2		
$Ni(OAc)_2 {*} 4H_2O$			0	0.5		
Bis(2-methylallyl)(1,5-cyclooctadiene)ruthenium(II)	2.05	7.85	0.2	10		
$TiCpCl_3$	0.00	50.80	0	74		
$ZrCl_4$	0.00	91.34	0.00	85.40		
$[Rh(COD)_2]BF_4$	0.00	7.26	0.00	4.64		
Dichloro(mesitylene)ruthenium(II) dimer	0.00	9.33				
Dichloro(benzene)ruthenium(II) dimer	0.77	7.89				
Dichloro(p-cymene)triphenylphosphineruthenium(II) dichloromethane adduct	0.62	7.31				
Dichloro(1,5-cyclooctadiene)ruthenium(II) polymer	0.45	8.55				
Cyclopentadienyl(p-cymene)ruthenium(II) hexafluorophosphate	0.00	4.62				
Tris(acetonitrile)pentamethylcyclopentadienylruthenium(II) hexafluorophosphate	0.00	68.73				
$Pd(OAc)_2$	0.25	9.21				

Table s9. Additional ligand screening for the vinylation reaction with vinyl acetate and selected Ru compounds

Catalyst	BzOVi	BzOAC	Ligand/additive
$Ru_2Cl_4Cymene_2$	0.52	2.64	**PPh_3**
	0.61	2.99	**pCF_3PPh_3**
	0.60	2.57	**Pyridine**
	0.79	2.37	**Adm-PPh**
	0.79	83.96	**Zn powder**
Bis(2-methylallyl)(1,5-cyclooctadiene)ruthenium(II)	0.52	3.00	**PPh_3**
	0.43	2.50	**pCF_3PPh_3**
	0.56	3.48	**Pyridine**
	0.78	2.45	**Adm-PPh**
	1.08	28.94	**Zn powder**

Table s10. Variable time screening for the vinylation reaction with vinyl acetate and selected Ru compounds

		**Overnight**		**50 min**
**Catalyst**	**BzOVi**	**BzOAC**	**BzOVi**	**BzOAC**
$Ru_2Cl_4Cymene_2$	0.80	6.94	0.45	1.97
$Ru_2Cl_4Cymene_2$ + Phenantroline	1.51	25.44		
Bis(2-methylallyl)(1,5-cyclooctadiene)ruthenium(II)	0.77	8.14	0.45	1.97
Bis(2-methylallyl)(1,5-cyclooctadiene)ruthenium(II) + Phenantroline	1.84	29.59		
Bis(2-methylallyl)(1,5-cyclooctadiene)ruthenium(II) + DPPE	0.92	11.70		

Table s11. 10 mol% catalyst loading for the vinylation reaction with vinyl acetate and selected Ru compounds

		**10 mol% cat. loading**
**Catalyst**	**BzOVi**	**BzOAC**
$Ru_2Cl_4Cymene_2$	2.06	9.71
Bis(2-methylallyl)(1,5-cyclooctadiene)ruthenium(II)	1.59	10.65

Table s12. Temperature screening for the vinylation reaction with vinyl acetate and selected Ru compounds

**Temperature**	**100 °C**		**120 °C**		**140 °C**	
**Catalyst**	**BzOVi**	**BzOAC**	**BzOVi**	**BzOAC**	**BzOVi**	**BzOAC**
$Ru_2Cl_4Cymene_2$	0	0	0.85	5.96	1.34	14.20
$Ru_2Cl_4Cymene_2$ + Phenantroline	0	23.5	0.96	6.73	2.20	37.35
Bis(2-methylallyl)(1,5-cyclooctadiene)ruthenium(II)	0	0	0.88	7.12	1.25	14.86
Bis(2-methylallyl)(1,5-cyclooctadiene)ruthenium(II) + Phenantroline	2.05	7.85	2.07	25.89	1.96	28.48
Bis(2-methylallyl)(1,5-cyclooctadiene)ruthenium(II) + DPPE	0.2	10	0.92	7.20	1.13	14.96
Bis(2,2,6,6-tetramethyl-3,5-heptanedionato)(1,5-cyclooctadiene)ruthenium(II)	0	0				
Hexakis[μ-(acetato-O:O')]-triaqua-μ3-oxotriruthenium(III); (Ruthenium(III) acetate)	0	0				
Dichloro(mesitylene)ruthenium(II) dimer	0.38	4.09				
Dichloro(benzene)ruthenium(II) dimer	0.73	3.36				
Dichloro(p-cymene)triphenylphosphineruthenium(II) dichloromethane adduct	0.46	2.42				
Dichloro(1,5-cyclooctadiene)ruthenium(II) polymer	0.00	2.18				
Cyclopentadienyl(p-cymene)ruthenium(II) hexafluorophosphate	0.00	5.19				
Tris(acetonitrile)pentamethylcyclopentadienylruthenium(II) hexafluorophosphate	0.00	1.77				

Table s13. Solvent (temperature) screening for the vinylation reaction with vinyl acetate and selected Ru compounds

**Solvent - Temperature**	**100 °C**	**2-MeTHF**	**120° C**	**Chlorobenzene**	**120 °C**	**Toluene**
**Catalyst**	**BzOVi**	**BzOAC**	**BzOVi**	**BzOAC**	**BzOVi**	**BzOAC**
$Ru_2Cl_4Cymene_2$	0	0	1.22	10.00	1.55	8.19
$Ru_2Cl_4Cymene_2$ + Phenantroline	0	23.5				
Bis(2-methylallyl)(1,5-cyclooctadiene)ruthenium(II)	0	0	1.22	8.20	1.21	7.21
Bis(2-methylallyl)(1,5-cyclooctadiene)ruthenium(II) + Phenantroline	2.05	7.85				
Bis(2-methylallyl)(1,5-cyclooctadiene)ruthenium(II) + DPPE	0.2	10				
Bis(2,2,6,6-tetramethyl-3,5-heptanedionato)(1,5-cyclooctadiene)ruthenium(II)	0	0				
Hexakis[µ-(acetato-O:O')]-triaqua-µ3-oxotriruthenium(III); (Ruthenium(III) acetate)	0	0				
Dichloro(mesitylene)ruthenium(II) dimer	0.38	4.09	1.16	9.07	1.22	7.01
Dichloro(benzene)ruthenium(II) dimer	0.73	3.36	1.53	9.32	1.63	6.55
Dichloro(p-cymene)triphenylphosphineruthenium(II) dichloromethane adduct	0.46	2.42	0.89	8.25	0.80	5.98
Dichloro(1,5-cyclooctadiene)ruthenium(II) polymer	0.00	2.18	0.99	7.97	0.95	6.24
Cyclopentadienyl(p-cymene)ruthenium(II) hexafluorophosphate	0.00	5.19	1.53	9.32	0.00	1.39
Tris(acetonitrile)pentamethylcyclopentadienylruthenium(II) hexafluorophosphate	0.00	1.77	0.00	2.53	0.00	1.47

Table s14. Base screening (30 mol%) for the vinylation reaction with vinyl acetate and selected Ru compounds

**Base/mol% (other additions)**		**NaOAC**		**Na_2CO_3**	**(Mol. sieve)**	**Na_2CO_3**
**Catalyst**	**BzOVi**	**BzOAC**	**BzOVi**	**BzOAC**	**BzOVi**	**BzOAC**
$Ru_2Cl_4Cymene_2$	0	0	0.99	9.72	0.95	16.21
Bis(2-methylallyl)(1,5-cyclooctadiene)ruthenium(II)	0	0	0.70	10.05	0.83	19.55
Dichloro(mesitylene)ruthenium(II) dimer	0.38	4.09	0.83	10.89	0.61	19.36
Dichloro(benzene)ruthenium(II) dimer	0.73	3.36	0.88	7.15	0.97	21.09
Dichloro(p-cymene)triphenylphosphineruthenium(II) dichloromethane adduct	0.46	2.42	0.92	4.91		
Dichloro(1,5-cyclooctadiene)ruthenium(II) polymer	0.00	2.18	0.52	5.10		
Cyclopentadienyl(p-cymene)ruthenium(II) hexafluorophosphate	0.00	5.19	0.00	8.33		
Tris(acetonitrile)pentamethylcyclopentadienylruthenium(II) hexafluorophosphate	0.00	1.77	0.00	3.85		

Table s15. Base screening for the vinylation reaction with vinyl acetate and selected Ru compounds

**Base/mol% (other additions)**		**No Base**	**20 %**	**Phenantroline**	**20 %**	**Et_3N**
**Catalyst**	**BzOVi**	**BzOAC**	**BzOVi**	**BzOAC**	**BzOVi**	**BzOAC**
$Ru_2Cl_4Cymene_2$	0.55	1.29	0.00	0.63	0.57	2.07
Bis(2-methylallyl)(1,5-cyclooctadiene)ruthenium(II)	0.80	3.36	0.00	52.45	0.37	4.28
Dichloro(benzene)ruthenium(II) dimer	0.62	0.74	0.00	0.62		

Table s16. Base screening for the vinylation reaction with vinyl acetate and selected Ru compounds

**Base/mol% (other additions)**	**20 %**	**Aniline**	**20 %**	**p-NO_2-Aniline**	**20 %**	**Proton sponge**
**Catalyst**	**BzOVi**	**BzOAC**	**BzOVi**	**BzOAC**	**BzOVi**	**BzOAC**
$Ru_2Cl_4Cymene_2$	0.00	0.00	0.00	0.00	0.91	1.68
Bis(2-methylallyl)(1,5-cyclooctadiene)ruthenium(II)	0.00	4.43	0.39	1.33	0.50	3.51
Dichloro(mesitylene)ruthenium(II) dimer			0.00	0.00	0.48	0.92
Dichloro(benzene)ruthenium(II) dimer			0.00	0.78	1.29	1.90

References

[1] M. Tesch, J. A. Hepperle, H. Klaasen, M. Letzel, A. Studer, *Angew. Chem. Int. Ed.* **2015**, *54*, 5054-5059.

[2] Y. Okimoto, S. Sakaguchi, Y. Ishii, *J. Am. Chem. Soc.* **2002**, *124*, 1590-1591.

[3] L. A. Oparina, S. I. Shaikhudinova, L. N. Parshina, O. V. Vysotskaya, T. Preiss, J. Henkelmann, B. A. Trofimov, *Russ. J. Org. Chem.* **2005**, *41*, 656-660.

[4] N.-a. Harada, T. Nishikata, H. Nagashima, *Tetrahedron* **2012**, *68*, 3243-3252.

[5] P. Hu, S. Huang, J. Xu, Z.-J. Shi, W. Su, *Angew. Chem. Int. Ed.* **2011**, *50*, 9926-9930.

[6] S. Matysiak, H.-P. Fitznar, R. Schnell, W. Pfleiderer, *Helv. Chim. Acta* **1998**, *81*, 1545-1566.

[7] S. L. Schreiber, R. A. Stavenger, Z. Maliga, T. J. Mitchison, (Howard Hughes Medical Institute), US20040059138A1, United States, **2004**.

[8] B. Spiegelberg, H. Jiao, R. Grauke, C. Kubis, A. Spannenberg, A. Brandt, A. Taden, H. Beck, S. Tin, J. G. Vries, *Adv. Synth. Catal.* **2022**, *364*, 1251-1263.
